# Rapid Antibody Structural Characterization and Quantification via Microdroplet Trypsin Digestion

**DOI:** 10.1021/jasms.5c00457

**Published:** 2026-02-15

**Authors:** Mengyuan Xiao, Yongqing Yang, Andrew Schladebeck, Jim Lau, Michael Knierman, Hui Zhao, Xi Qiu, Karen Luo, Harsha P. Gunawardena, Hao Chen

**Affiliations:** † Department of Chemistry & Environmental Science, 5965 New Jersey Institute of Technology, Newark, New Jersey 07102, United States; ‡ Agilent Technologies, Inc., 2850 Centerville Rd, Wilmington, Delaware 19807, United States; § Johnson & Johnson Innovative Medicine, Johnson & Johnson, 1400 McKean Road, Spring House, Pennsylvania 19477, United States

## Abstract

Microdroplet digestion of proteins, including monoclonal antibodies (mAbs), has gained increasing attention for its potential in rapid protein structural characterization. In this study, we developed a fast antibody characterization and quantitation method using online microdroplet trypsin digestion. This automated approach involves ultrafast digestion in <1 ms (>90% digestion efficiency) and subsequent MS and MS/MS for peptide mapping and pinpointing peptide modifications. For instance, by MS/MS analysis, the N^387^ residue rather than N^392^ and N^393^ of the “PENNYK” peptide, GFYPSDIAVEWESN^387^GQPEN^392^N^393^YK generated from NIST mAb microdroplet digestion, was found to undergo major deamidation due to its proximity to the G^388^ residue (upon incubation with a pH 8 Tris buffer for 5 days, ca. 32% of the N^387^ residue was deamidated). To further demonstrate our method’s applications, methionine oxidation, another important type of post-translational modification (PTM) of mAbs, was successfully quantified by spiking the mAb sample with standard peptides for microdroplet digestion. Such an absolute quantitation approach showed a better accuracy for measuring the methionine oxidation level, in comparison to traditional relative quantitation methods, which simply compare ion intensities of oxidized and intact peptides to calculate the oxidation level (the relative quantitation is problematic as the oxidized and intact peptides have different ionization efficiencies). Similarly, by spiking the antibody with a heavy isotope-labeled antibody, our microdroplet digestion method allowed quick absolute antibody quantification, demonstrating good linearity (*R* ^2^ = 0.99), sensitivity (LOD: 1.2 ng), and accuracy (0.6–10% quantification error in comparison to the theoretical amount of injected antibody).

## Graphical abstract

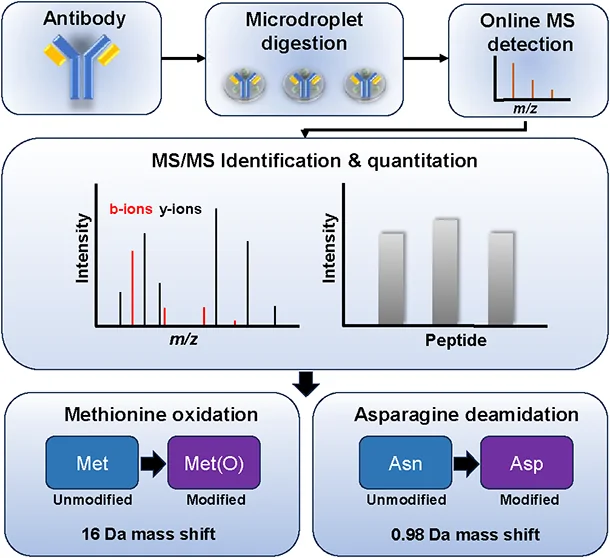

## Introduction

Microdroplet is an unique reaction media known to speed up reaction rates significantly, in comparison to the same reaction in solution.^1–6^ Many reactions can be accelerated when being carried out in nebulized microdroplets with a reaction time less than 1 ms.^1,2,5–9^ Previously, we have reported both offline and online microdroplet digestion of therapeutic monoclonal antibodies (mAbs)^4,10–12^ via mixing and spraying mAbs together with proteolytic enzymes such as trypsin and Ides, from which we successfully detected antigen-binding F­(ab′) subunits and single-chain fragment crystallizable (scFc) subunits carrying different glycoforms. Microdroplets produced via nebulization generate ions and thus allow the detection of microdroplet reaction products by mass spectrometry (MS).^13–17^ By utilizing the Agilent Jet Stream (AJS) Source, we showed that FabRICATOR Xtra enzyme and mAbs carrying several different Fc mutations “LALA”, “LAGA”, and “LFLE” were shown to be efficiently digested in microdroplets and achieved online MS characterization.^4^ Online deglycosylation of reduced antibodies in microdroplets using enzyme PNGase F achieved 100% digestion efficiency and complete sequence coverage based on MS results, suggesting great potential for applications in the pharmaceutical industry for analyzing glycan modifications on subunits.^10^ Online microdroplet digestion mass spectrometry was also coupled with liquid chromatography (LC) for protein mixture analysis.^8,9^ As demonstrated by Ma et al., protein mixture was separated by LC with acidic mobile phase and online digested in microdroplet by pepsin which was introduced post column.^9^ However, pepsin digestion generates many unspecific cleavages that are difficult to analyze. Trypsin digestion is more popular because it produces highly specific cleavages on C terminals of lysine and arginine.^18^ We reported the offline microdroplet digestion of the antibody using trypsin and successfully performed peptide mapping analysis.^12^ The result showed less deamidation for peptides generated from microdroplet digestion compared with in-solution digestion. This phenomenon is critical to the antibody quality analysis, as artificial peptide deamidation should be minimized during the sample preparation step. However, so far, no study has been carried out to characterize and quantify post-translational modifications (PTMs) such as methionine oxidation and asparagine deamidation present in antibodies. Each deamidation introduces a 0.984 Da mass shift that is difficult to detect in complex spectra, while the presence of multiple methionine residues across the antibody sequence complicates oxidation analysis without detailed peptide-level characterization.^19,20^

PTMs of mAbs are important product quality attributes that can potentially impact drug stability, safety, and efficacy.^21–25^ It is important to characterize and quantify the PTMs of the antibody to ensure antibody drug quality. Traditionally, antibody was digested in solution by trypsin using a bottom-up LC-MS/MS approach.^21,26–30^ However, in-solution trypsin digestion is time-consuming and typically takes hours to overnight to perform,^30^ and can also lead to undesired solution-phase artifacts such as deamidation products.^31–34^ Advancement in the field of proteomics has demonstrated a number of approaches to minimize the digestion times during sample preparation and to automate analysis.^35,36^ Despite these advancements, rapid characterization of proteins, including antibodies, without the need for in-solution digestion is desirable.

In this study, we developed an ultrafast online microdroplet digestion method with a digestion time of less than 1 ms, representing a million-fold acceleration compared to conventional in-solution digestion. The microdroplet digestions are fully automated for uninterrupted sample introduction that enables real-time peptide mapping. This approach achieved a digestion efficiency of >90% by evaluating the ion intensity change of the antibody before and after digestion. Using iterative MS/MS, we obtained peptide maps with high sequence coverage for the reduced NIST and Bevacizumab mAbs. More importantly, PTMs, including asparagine deamidation and methionine oxidation, were effectively characterized with MS/MS acquisition and quantified using the standard addition method in combination with microdroplet digestion.^37^ We further demonstrated the linear relationship between ion intensity ratios of heavy to light peptide pairs and the injection amount of the spiked heavy isotope-labeled antibody from online microdroplet trypsin digestion of a target antibody after being spiked with the heavy antibody standard, thus allowing fast absolute antibody quantitation.

## Experimental Section

### Reagents

Ammonium bicarbonate (BioUltra Product No. 09830), iodoacetamide (Product No. I1149), dithiothreitol (Product No. D0632), hydrogen peroxide solution (Product No. H1009), NIST mAb Monoclonal Antibody Reference Standard 8617 (sequence shown in Scheme S1), SILuLite SigmaMAb Bevacizumab Monoclonal Antibody (i.e., light Bevacizumab mAb, Product No. MSQC20, sequence shown in Scheme S2), and SILuMAb Bevacizumab Stable-Isotope-Labeled Monoclonal Antibody (i.e., heavy isotope-labeled Bevacizumab mAb, Product No. MSQC21, sequence shown in Scheme S2) were purchased from Sigma-Aldrich (ST. Louis, MO). Tris­(2-carboxyethyl)­phosphine hydrochloride (TCEP, Product No. 20492) was purchased from Thermo Fisher Scientific (Ward Hill, MA). Trypsin (proteomics grade, Product No. 03708969001) was purchased from Roche (Mannheim, Germany). Peptide standard DTLMISR and oxidized DTLM­(O)­ISR were synthesized by RS synthesis (99.95% purity, Louisville, KY).

#### Sample Preparation for Reduced and Alkylated NIST mAb

For the sample preparation of reduced mAbs, NIST IgG1 was diluted to 1 mg/mL in 8 M urea containing 50 mM ammonium bicarbonate (ABC) buffer. Dithiothreitol (DTT) was added to a final concentration of 6 mM, and the solution was incubated at 37 °C for 1 h to reduce disulfide bonds. Subsequently, iodoacetamide (IAA) was added to a final concentration of 18 mM, and the mixture was incubated in the dark at room temperature for 30 min to alkylate the free thiol groups of the cysteine residues. Both the reduced and alkylated NIST IgG1 and trypsin were buffer-exchanged into 50 mM ABC using 10 kDa molecular weight cutoff filters. The NIST IgG1 was reconstituted at 0.2 mg/mL in 50 mM ABC, and trypsin was reconstituted at 0.1 mg/mL in 0.01% formic acid. After reconstitution, both solutions were stored separately at 6 °C in the autosampler for subsequent LC-MS flow injection analysis. Alternatively, for fast mAb reduction, 20 μL of NIST IgG stock solution (10 mg/mL) was diluted to 1 mg/mL with 5 mM ABC buffer. Then, 1 μL of 300 mM TCEP was added to the IgG solution for incubation at 60 °C for 5 min.

#### Online Microdroplet Digestion

All experiments were performed on an Agilent 1290 Infinity II LC instrument coupled to an Agilent 6545XT Q/TOF mass spectrometer. Prior to the experiment, the instrument was calibrated in high-resolution mode with a 4 GHz dynamic range and a standard mass range setting of 50–3200 *m*/*z*. Antibody and sequencing-grade trypsin, stored in a LC autosampler at 6 °C, were serially aspirated, mixed in an injection loop, and injected into the flow path using an injector program (shown in Table S1), as described previously.^4^ A 2 min flow injection (FI) carried the mixture at 100 μL/min with the mobile phase solvent of 5 mM ammonium bicarbonate (ABC) buffer into an Agilent Jet Stream (AJS) ion source, generating microdroplets. For each injection, 0.2 μL of NIST mAbs (1 μg/μL) and 0.2 μL of trypsin (0.1 μg/μL) were aspirated, resulting in a protein-to-enzyme ratio of 10:1 during microdroplet digestion. Iterative tandem mass spectrometry (MS/MS) was utilized to enhance the data acquisition depth and protein sequence coverage. The protein and enzyme were mixed and injected multiple times, with previously selected precursor ions dynamically excluded in each iteration, ensuring greater peptide ion analysis from the microdroplet digestion.

#### Digestion Efficiency Comparison

A 200 μL aliquot of 1 mg/mL NIST mAb in 5 mM ABC buffer was reduced by adding 2 μL of 300 mM TCEP and incubating at 60 °C for 5 min. The reduced antibody was then desalted and buffer-exchanged into 5 mM ABC using a 3 kDa molecular weight cutoff filter (MWCO) filter and reconstituted to a final concentration of 0.5 mg/mL. The sample was divided into two portions for comparison of online microdroplet digestion and in-solution digestion. For in-solution digestion, 50 μL of the reduced mAb was mixed with 50 μL of 0.1 mg/mL trypsin and incubated at 47 °C for 2 h. For online microdroplet digestion, 50 μL of reduced mAb was diluted with 50 μL of 5 mM ABC buffer. Standard DTLMISR and oxidized DTLM­(O)­ISR peptides were prepared in deionized water (DIW) at 0.31 mg/mL and diluted 25×, 50×, 100×, 200×, and 500× to generate working solutions. For both workflows, 10 μL of each peptide standard solution was spiked into 10 μL of reduced mAb (for microdroplet digestion) or in-solution-digested mAb (for in-solution comparison) individually. Online microdroplet digestion was performed by injecting 0.2 μL of reduced mAb mixed with 0.2 μL of 0.1 mg/mL trypsin using a programmed flow injection. In contrast, for in-solution digestion, 0.2 μL of the digested mAb was injected after mixing with 0.2 μL of deionized water using programmed flow injection for MS analysis.

#### Sample Preparation and Quantitation for Hydrogen Peroxide-Stressed NIST mAb

A 20 μL aliquot of 10 mg/mL NIST mAb stock solution was diluted with 180 μL of 0.1% (v/v) hydrogen peroxide (H_2_O_2_) and mixed using a vortex at room temperature. After 1 h of vortexing, 50 μL of the stressed sample was transferred to a new vial, followed by adding 1 μL of 100 mM TCEP solution, and incubated at 60 °C for 5 min to reduce the oxidized mAbs. The reduced samples were analyzed by online microdroplet digestion coupled with targeted MS/MS monitoring of DTLMISR (*m*/*z* 418.22, +2) and oxidized DTLM­(O)­ISR (*m*/*z* 426.22, +2). The same procedure was repeated after incubation of mAbs with H_2_O_2_ for 0, 2, and 3 h, to evaluate the peptide oxidation over time.

The standard DTLMISR peptide and its oxidized form were prepared in deionized water at a concentration of 0.31 mg/mL as stock solutions. These stocks were subsequently diluted 25×, 50×, 100×, 200×, and 500× to generate a series of working concentrations. For the nonstressed NIST mAb, 100 μL of 1 mg/mL NIST mAb in 5 mM ABC buffer was reduced by incubating with 1 μL of 300 mM TCEP at 60 °C for 5 min. Then, 10 μL of each DTLMISR standard solution was spiked into 10 μL of the reduced antibody. Online microdroplet digestion was performed by mixing 0.2 μL of the spiked NIST mAb with 0.2 μL of 0.1 mg/mL trypsin by using programmed flow injection. For hydrogen peroxide-stressed samples, the stressed antibody was then reduced with 1 μL of 300 mM TCEP at 60 °C for 5 min. Next, 10 μL of either the DTLMISR or DTLM­(O)­ISR standard solution was spiked individually into 10 μL of the stressed mAb. Online microdroplet digestion was carried out in the same way as the procedure used for the nonstressed NIST mAb.

#### Sample Preparation for Deamidated NIST mAb

20 μL of NIST mAbs stock solution (10 mg/mL) was diluted 10 times with water or 1 M pH 8 Tris buffer and served as control and deamidated samples, respectively. The diluted mAbs were incubated at 40 °C for 5 to 7 days. After incubation, 1 μL of 300 mM TCEP was used to both sample and incubate at 60 °C for 5 min to reduce the mAbs. Buffer exchange of both samples was performed with a 10K MWCO filter to a 5 mM ABC buffer. During the microdroplet digestion, different asparagine-containing peptides were monitored by target MS/MS with a collisional energy (CE) of 15 eV, to check the occurrence of their asparagine residue's deamidation levels.

#### Absolute Quantification of mAb Using Microdroplet Digestion

For absolute antibody quantification, light- and heavy-isotope-labeled Bevacizumab mAbs were first dissolved in deionized water at 1 mg/mL according to the vendor-provided mass information. Each antibody solution was then buffer-exchanged into 5 mM ABC using a 10 kDa MWCO centrifugal filter to remove residual salts from the original formulation. After buffer exchange, the antibodies were reconstituted to a final concentration of 0.5 mg/mL. To reduce the antibodies, 1 μL of 300 mM TCEP was added to 150 μL of each desalted antibody solution, followed by incubation at 47 °C for 30 min. For the preparation of calibration mixtures, 2.5 μL of light Bevacizumab mAb was combined with varying amounts of heavy isotope-labeled Bevacizumab mAb, and the volume was adjusted to 20 μL with deionized water. The theoretical concentration of light Bevacizumab mAb was calculated to be 0.625 mg/mL. Microdroplet digestion of the Bevacizumab mAbs was performed online using 10 mM ABC as the mobile phase at a flow rate of 20 μL/min with a 0.2 μL injection volume. So, the injected amount of light antibody was calculated to be 12.5 ng. Q-TOF acquisition parameters were identical to those used for microdroplet digestion of the NIST mAb.

### Instrumentation

#### Agilent Jet Stream (AJS) Ionization Source

The AJS drying gas flow rate and sheath gas temperature were set at 12 L/min and 350 °C. The nebulizer nitrogen gas was operated at 60 psi. The mass spectrometer TOF analyzer was optimized with the Fragmentor set to 130 V, Skimmer set to 65 V, VCap voltage set to 3500 V, and Nozzle voltage set to 500 V for all experiments. MS1 spectra were collected between 250–3200 *m/z*, with a collection rate of 6 spectra/s. MS/MS spectra were collected between 100 and 1700 *m*/*z*, with a collection rate of 4 spectra/s with an isolation window of 4 Da. A 4 Da window is generated by the following formula

centerm/z=(monoisotopicprecursorm/z−0.3)±2

#### Iterative MS/MS

Due to the complexity of antibody peptides generated via microdroplet digestion, MS/MS acquisition parameters were optimized for microdroplet digestion, which could select many precursor ions in 30 s time period during the flow injection. For auto MS/MS precursor selection, only 2 and 3 charged ions were targeted for analysis, with an MS/MS scan rate of 4 spectra per second. A maximum of 5 precursors were analyzed per cycle, and each precursor was excluded after being scanned twice. The collision energy for 2 charged peptides was set to CE = 3.1*­(*m*/*z*)/100 + 1, and for 3 charged peptides, the collisional energy was set to CE = 3.6*­(*m*/*z*)/100–4.8. The precursor fragmentation was sorted for picking up the corrected charge states (+2 and +3 charges) and then sorted by the abundance. Iterative MS was performed with three continuous injections under identical parameters.

## Results and Discussion

### Microdroplet Digestion of Reduced NIST Antibody

The general workflow for online microdroplet digestion of NIST mAb is described in Figure 1a. Prior to microdroplet digestion, NIST mAb was reduced in solution using TCEP and alkylated using IAA, which is a critical step to break the inter- and intrachain disulfide bonds, promote protein unfolding, enhance enzymatic accessibility, and reduce peptide identification complexity. Following reduction and alkylation, the mAb sample was desalted using a MWCO filter to remove excess reagents and salts. In the autosampler, the prepared mAb solution and trypsin enzyme solution were placed in separate vials. Through a programmed flow injection sequence (Figure S1), the mAb and trypsin solutions were aspirated and mixed in the injection needle. This mixture was then carried to the AJS ion source with 5 mM ABC buffer and nebulized into microdroplets for digestion, and the digested mAb was immediately detected by the Q-TOF mass spectrometer. Three iterative MS/MS analyses were done to increase the MS/MS identification depth. As shown in Figure 1b, the reduced antibody produced strong ion signals corresponding to charge state distributions of the light chain (LC) and heavy chain (HC) in the 800–2400 *m*/*z* range when injected without trypsin (deconvoluted spectra shown in Figure S1a,c). When trypsin and mAb were coinjected using the programmed autosampler, the antibody underwent online digestion in microdroplets, and the resulting tryptic peptides appeared in the *m*/*z* 300–1300 region (Figure 1c), accompanied by a substantial signal decrease for light- and heavy-chain ions. Deconvoluted spectra (Figure S1) showed that the light-chain signal decreased from 7.2 × 10^5^ to 7.0 × 10^4^ and the heavy-chain signal decreased from 8.8 × 10^4^ to 8.1 × 10^2^ following digestion (Figure S1a–d), corresponding to digestion efficiencies of approximately 90% for the light chain and 99% for the heavy chain (Figure S1e), respectively.

**Figure 1:**
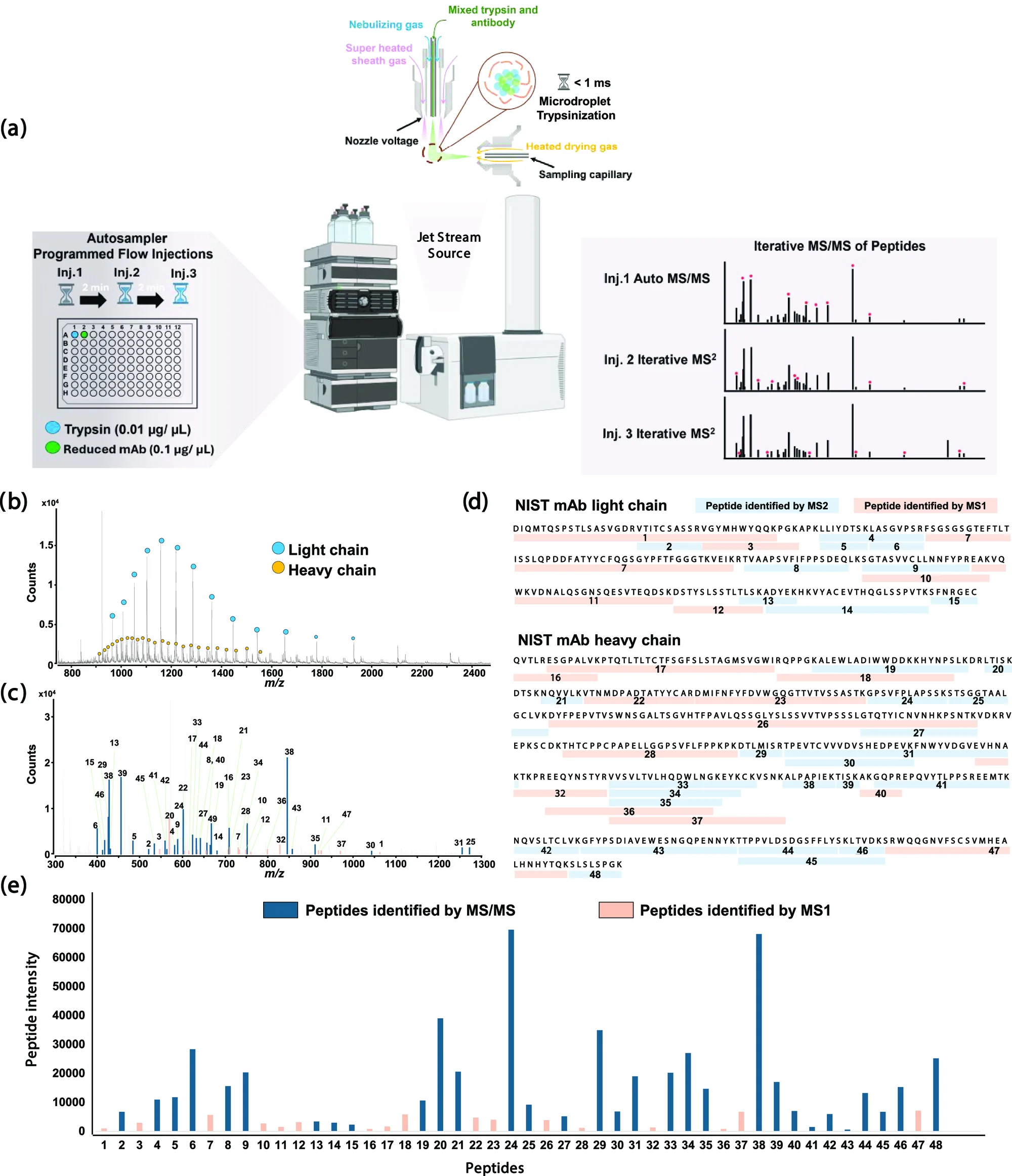
(a) Scheme illustrating microdroplet digestion of the antibody with trypsin performed with programmed flow injection via an autosampler injector of the LC system coupled with a Q-TOF mass spectrometer with iterative auto MS/MS acquisition. MS spectra of (b) reduced NIST mAb before microdroplet digestion and (c) reduced mAb after microdroplet digestion; (d) peptide identification map for NIST mAb with MS and MS/MS analyses; and (e) plot showing the peak intensities for identified peptides.

As shown in Figure 1d, 53% sequence coverage was achieved by three replicates of iterative MS/MS. 84% of the MS/MS identified peptides had an intensity higher than 5000 counts (Figure 1e), which enabled higher confidence of identification, while the low intensity peptide ions were identified by MS1. The overall sequence coverage achieved 91% when combining both MS and MS/MS identifications.

Microdroplet digestion efficiency was further evaluated against traditional in-solution digestion using a standard addition approach. As shown in Figure S2, standard addition curves were generated by spiking varying concentrations of the standard DTLMISR, one NIST mAb tryptic peptide, into reduced NIST mAb samples subjected either to microdroplet digestion or traditional in-solution digestion. The two curves exhibited similar slopes and intercepts. The concentrations of DTLMISR generated from NIST mAb digestion, obtained from the x-intercept equals to zero, were calculated to be 0.54 μg/mL (6.47 × 10^–7^ mol/L) for microdroplet digestion and to be 0.59 μg/mL (7.07 × 10^–7^ mol/L) for in-solution digestion. The former concentration is slightly lower than the latter concentration, suggesting that the microdroplet digestion has a close digestion efficiency with in-solution digestion. The theoretical initial concentration of the NIST antibody was 8.45 × 10^–7^ mol/L, which would produce 1.69 × 10^–6^ mol/L of DTLMISR peptide if the protein was 100% digested (one NIST mAb contains two identical heavy chains carrying the peptide DTLMISR). This result suggests a relatively low yield of 38% for generating DTLMISR peptide from the microdroplet digestion of NIST mAb, which may be due to sample loss during desalting or incomplete digestion.

### Monitoring the Deamidation Level of NIST mAb

Deamidation is a critical PTM of an antibody because it can affect stability, pharmacokinetics, and stability of the antibody drug.^20,38,39^ Deamidation is a common spontaneous chemical reaction where the amide groups in asparagine (Asn) and glutamine (Gln) residues are converted to carboxylic acids. As a result, each deamidation introduces a 0.984 Da mass shift to a target peptide. Therefore, developing an efficient and accurate method to monitor deamidation is essential for controlling this modification during antibody production and storage.^40^ As shown in Figure 1d, five asparagine-containing peptides were identified by MS/MS performed on NIST mAb, including NQVVLK (No. 21 peptide, *m*/*z* 700.43, +1), TPEVTCVVVDVSHEDPEVKFNWYVDGVEVHNAK (No. 31 peptide, *m*/*z* 1247.60, +3), VVSVLTVLHQDWLNGKEYK (No. 34 peptide, *m*/*z* 743.40, +3), GFYPSDIAVEWESNGQPENNYK (No. 43 peptide, *m*/*z* 848.71, +3), and SGTASVVCLLNNFYPR (No. 9 peptide, *m*/*z* 870.94, +2). Artificial deamidation was introduced by incubating the NIST mAb in 1 M Tris buffer (pH 8) for 5 or 7 days. For comparison, the control sample was made by incubating the same antibody in deionized water (neutral pH), with the same durations. Target MS/MS was further performed on these five peptide ions during online microdroplet digestion for deamidation monitoring. Our calculation for the peptide deamidation percentage is shown in eq 1

1 $$\%\;\mathrm{deamidation}=\left(I_{y+1}-I_{y}\times \alpha \right)/\left(I_{y+1}-I_{y}\times \alpha +I_{y}\right)\times 100\%$$

where ***I***_*y*_ is the measured intensity of the monoisotopic peak of the *y* fragment ion and ***I***_*y*+1_ is the intensity of the *y* + 1 peak (i.e., the M + 1 isotope peak of the *y* fragment ion); α = theoretical ratio between y + 1 peak height and y ion monoisotopic peak height of the nondeamidated peptide.

As shown in Figure S3a, the MS spectrum exhibited weak intensity for the +3 ion (5.0 × 10^2^ counts, *m*/*z* 848.71) of peptide GFYPSDIAVEWESNGQPENNYK generated from microdroplet digestion of NIST mAb after 5 days of incubation in deionized water, and the signal was partially overlapped with background interference. Moreover, microdroplet-digested NIST mAb incubated in Tris buffer produced an even lower ion signal with a high background noise (2.2 × 10^2^ counts, Figure S3b). Due to the low MS signal intensity and background interference, accurate quantification of deamidation at the MS1 level was not feasible. Thereby, targeted MS/MS was performed on *m*/*z* 848.71 using a 4 Da isolation window, and its fragment ions were used for the deamidation evaluation. As shown in Figure 2a, the target MS/MS shows the fragmentation of the +3 ion of GFYPSDIAVEWESNGQPENNYK peptide (*m*/*z* 848.71), yielding fragment ions of a series of *y*_1_–*y*_11_ ions except *y*_9._ Among these fragments, *y*_10_ and *y*_6_ were selected to monitor the deamidation level at specific asparagine residues for this peptide. As shown in Figure 2b, after 5 days of incubation, the *y*_6_ ion exhibited identical isotopic distributions under the control and deamidation conditions, with the averaged peak height ratio of *y*_6_ + 1 to *y*_6_ as 0.42 for the control condition and 0.42 for the deamidated condition. The measured peak ratios of *y*_6_ + 1 to *y*_6_ were just slightly higher than the theoretical *y*_6_ + 1 to *y*_6_ ratio of 0.36 (Table S2), indicating that minimum deamidation occurred at both N^392^ and N^393^ residues. In contrast, Figure 2c shows that the ion intensities of *y*_10_ + 1 isotopic peaks significantly increased, with the averaged peak height of *y*_10_ + 1 to *y*_10_ as 0.72 for the control condition and 1.15 for the deamidated condition. This shift in the isotopic peak distribution strongly suggests that deamidation occurred primarily at N^387^, rather than at N^392^ or N^393^. Based on the specific isotopic peak distribution shifts observed for the *y*_6_ and *y*_10_ ions, the relative deamidation percentage of the peptide GFYPSDIAVEWESNGQPENNYK (Figure 2d,e) was quantified by dividing the increased height of the *y* + 1 peak (i.e., observed *y* + 1 peak height minus the theoretical *y* + 1 peak height without deamidation) by the sum of the increased height of *y* + 1 peak and the measured monoisotopic peak height of *y* ion, as shown in eq 1 (detailed calculation results shown in Table S2). As shown in Figure 2b,2c, the theoretical isotopic distribution of the fragment ions *y*_6_ and *y*_10_ was simulated based on their molecular formulas C_33_H_50_N_9_O_12_^+^ and C_47_H_72_N_15_O_19_^+^, respectively. As shown in Figure 2d, with 5-day incubation calculated based on *y*_6_ ion, the peptide from the antibody incubated in both deionized water and tris buffer exhibited low deamidation levels of 5 and 7%, respectively, at N^392^ and N^393^. It can be seen that residues N^392^ and N^393^ are not prone to be deamidated. In contrast, with 5-day incubation calculated based on *y*_10_ ion, the deamidation percentage changed to 15 and 39% for the antibody incubated in deionized water and tris buffer, respectively. It can be seen that the N^387^ residue underwent 10 and 32% deamidation for the antibody incubated in deionized water and tris buffer, respectively. The quantification results illustrate that the deamidation is more likely to occur to N^387^ (NG motif) rather than to N^392^ and N^393^ (NN motif). Our result matches with the previous study on asparagine deamidation propensity where asparagine is more likely to undergo deamidation when next to glycine, and less likely to undergo deamidation when next to another asparagine.^41,42^ Based on the 3D structure, the asparagine next to glycine has greater conformational freedom to adopt the geometry required to form succinimide and has lower activation energy to start the deamidation reaction. Notably, when the antibody was incubated in the Tris buffer for 7 days (Figure 2f), quantification based on the *y*_*6*_ ion showed an increase in deamidation to approximately 11%, indicating an additional ∼4% deamidation at N^392^ and N^393^ compared to 5 days. Meanwhile, *y*_10_-based quantification increased to 45%, reflecting an additional 6% deamidation for all three asparagines after extended incubation. The slightly lower average deamidation observed from the *y*_10_ ion in the 7-day control sample compared to the 5-day control is likely due to instrumental or systematic variability.

**Figure 2:**
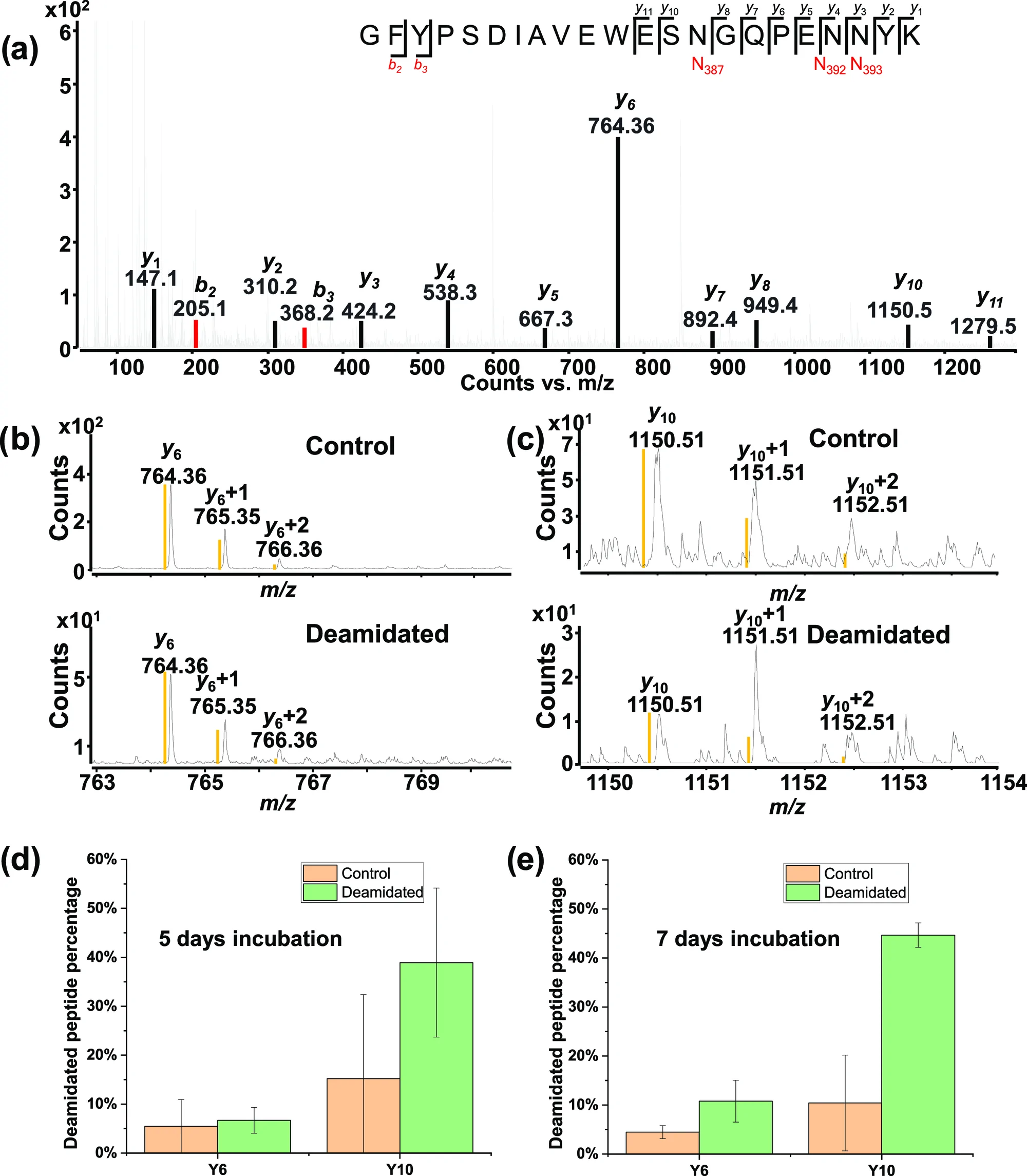
(a) MS/MS spectrum of +3 ion of GFYPSDIAVEWESNGQPENNYK (*m*/*z* 848.71) generated from microdroplet digestion of NIST antibody. Zoomed-in MS spectra showing (b) *y*_6_ fragment ion and (c) *y*_10_ fragment ion of *m*/*z* 848.71 resulting from online microdroplet digestion of NIST mAb incubated in deionized water (control) versus in 1 M Tris buffer (pH 8, deamidation condition) for 5 days, showing the minimum deamidation on N^392^ and N^393^; theoretical isotopic distribution patterns of the fragment ions are shown in yellow for comparison; *y*_10_ fragment ion of deamidated GFYPSDIAVEWESNGQPENNYK shows a higher *y*_10_ + 1 peak intensity over the *y*_10_ monoisotopic peak, indicating the occurrence of deamidation to N^387^. The deamidation percentages of peptide GFYPSDIAVEWESNGQPENNYK were calculated for NIST mAb incubated in deionized water and in Tris buffer for (d) 5 days and (e) 7 days, based on quantitative analysis of the *y*_6_ and *y*_10_ fragment ions.

Deamidation of the peptide TPEVTCVVVDVSHEDPEVKFN^279^WYVDGVEVHN^289^AK was also successfully monitored by online microdroplet digestion and MS/MS analysis. As shown in Figure S4a,b, both *y*_8_ and *y*_15_ ions showed an increased height of *y* + 1 peak over the monoisotopic peak of *y* ion, suggesting the deamidation could occur on both N^279^ and N^289^under deamidation condition. However, after checking the *b*_21_ fragment ion, the ratio of the *b*_21_ + 1 peak over the *b*_21_ monoisotopic peak was similar for mAb incubated in deionized water and tris buffer (Figure S4c), suggesting a minimum deamidation of N^279^, and the major deamidation occurred on N^289^. Based on *y*_8_ ion analysis using eq 1, approximately 8.2% of the peptide was deamidated after 7 days of incubation in Tris buffer, which is consistent with the 8.8% deamidation level estimated from the *y*_15_ ion (Table S3), further confirming N^289^ as the primary site of deamidation for this peptide. This is consistent with a previous report that the NA motif has a higher chance to be deamidated than the NW motif, due to the similar reason that alanine is less bulky than tryptophan.^41,42^ For peptide VVSVLTVLHQDWLN^318^GKEYK (shown in Figure S5 and Table S4), the *y*_9_ fragment was selected for deamidation monitoring, and the deamidation amount was calculated to be 4 and 10% under incubation in deionized water and tris buffer, respectively. For peptide N^78^QVVLK (Table S5), slight deamidation occurred at N^78^, which showed a similar deamidation percentage (3.7–4.3%) for incubation in both deionized water and tris buffer conditions. For peptide SGTASVVCLLN^136^N^137^FYPR (Table S6), after 7 days of incubation in tris buffer, the *y*_6_ fragment ion showed 13.6% deamidation, whereas the *y*_5_ fragment ion showed only 6.5% deamidation. These results indicate that N^136^, located in an LN motif, is more susceptible to deamidation than N^137^ located in an NF motif, consistent with the greater propensity for deamidation when asparagine is followed by a smaller residue such as leucine rather than a larger residue such as phenylalanine.^41,42^

### Quantification of Methionine Oxidation

The current gold standard for antibody oxidation quantification is bottom-up LC–MS/MS peptide mapping, which reliably measures oxidation levels at each individual methionine residue.^18^ By implementing online microdroplet digestion, our method eliminates the time-consuming in-solution digestion step and minimizes oxidation artifacts introduced during sample preparation. As shown in Figure S6, we compared the relative oxidation levels obtained from in-solution digestion versus those obtained from online microdroplet digestion. Using the peak intensities of oxidized peptide DTLM­(O)­ISR and unoxidized DTLMISR given in eq 2, we observed 4.7% oxidation for microdroplet digestion compared with 6.1% for in-solution digestion. In-source oxidation was also tested and was found to be negligible. This was confirmed by the fact that the oxidized peptide DTLM­(O)­ISR ion was less than 1% of that of intact DTLMISR (Figure S7) when the standard DTLMISR peptide was injected with the same acquisition method as microdroplet digestion. The result shows that 4.7% oxidation for DTLMISR occurred prior to the microdroplet digestion. The slightly higher oxidation level of 6.1% measured from in-solution digestion could be due to the additional oxidation occurring during the longer in-solution digestion process. This phenomenon was in agreement with our previous observation.^12^ Based on the 3D structure of IgG1 antibodies, the DTLMISR peptide lies near the neonatal Fc Receptor (FcRn) binding interface and is high solvent-exposed, making its methionine residue one of the most oxidation-sensitive sites in IgG1.^43^

2 $$\%\;o\mathrm{xidation}=I_{\mathrm{ox}}/\left(I+I_{\mathrm{ox}}\right)\times 100\%$$

where ***I***_ox_ is the DTLM­(O)­ISR ion (or fragment ion) intensity and ***I*** is the DTLMISR ion (or fragment ion) intensity.

3 $$\%\;o\mathrm{xidation}=C_{\mathrm{ox}}/\left(C_{\mathrm{ox}}+C\right)\times 100\%$$

where *C*_ox_ is the molar concentration of DTLM­(O)­ISR and *C* is the molar concentration of intact DTLMISR.

Developing accurate and artifact-minimized methods for quantifying oxidation of methionine residues is a particularly important critical quality attribute (CQA) of an antibody. As shown in Figure 3a, microdroplet digestion of hydrogen peroxide-stressed NIST mAbs produced both the native DTLMISR peptide ion (+2, *m*/*z* 418.22) and its oxidized counterpart DTLM­(O)­ISR (+2, *m*/*z* 426.22) in the MS spectrum. The 16 Da mass shift between the two ions indicates methionine oxidation to form methionine sulfoxide (the oxidation product with a 32 Da mass shift was not observed, indicating that dual oxidation of the methionine residue to form methionine sulfone did not occur). Targeted MS/MS was then performed on *m*/*z* 418.22 and *m*/*z* 426.22 using a 4 Da isolation window. As shown in Figure 3b,3c, the *y*_4_, *y*_5_, and *y*_6_ ions each exhibited a 16 Da increase in the oxidized form (while the *y*_3_ ion has no mass shift), further confirming that oxidation occurred at the methionine residue. Note that a 4 Da isolation window could also include other neighboring peptide ions for ion dissociation. For example, the peaks in yellow were fragment ions from the +2 ion of ALPAPIEK, which was cofragmented with the DTLMISR ion (Figure 3b).

**Figure 3:**
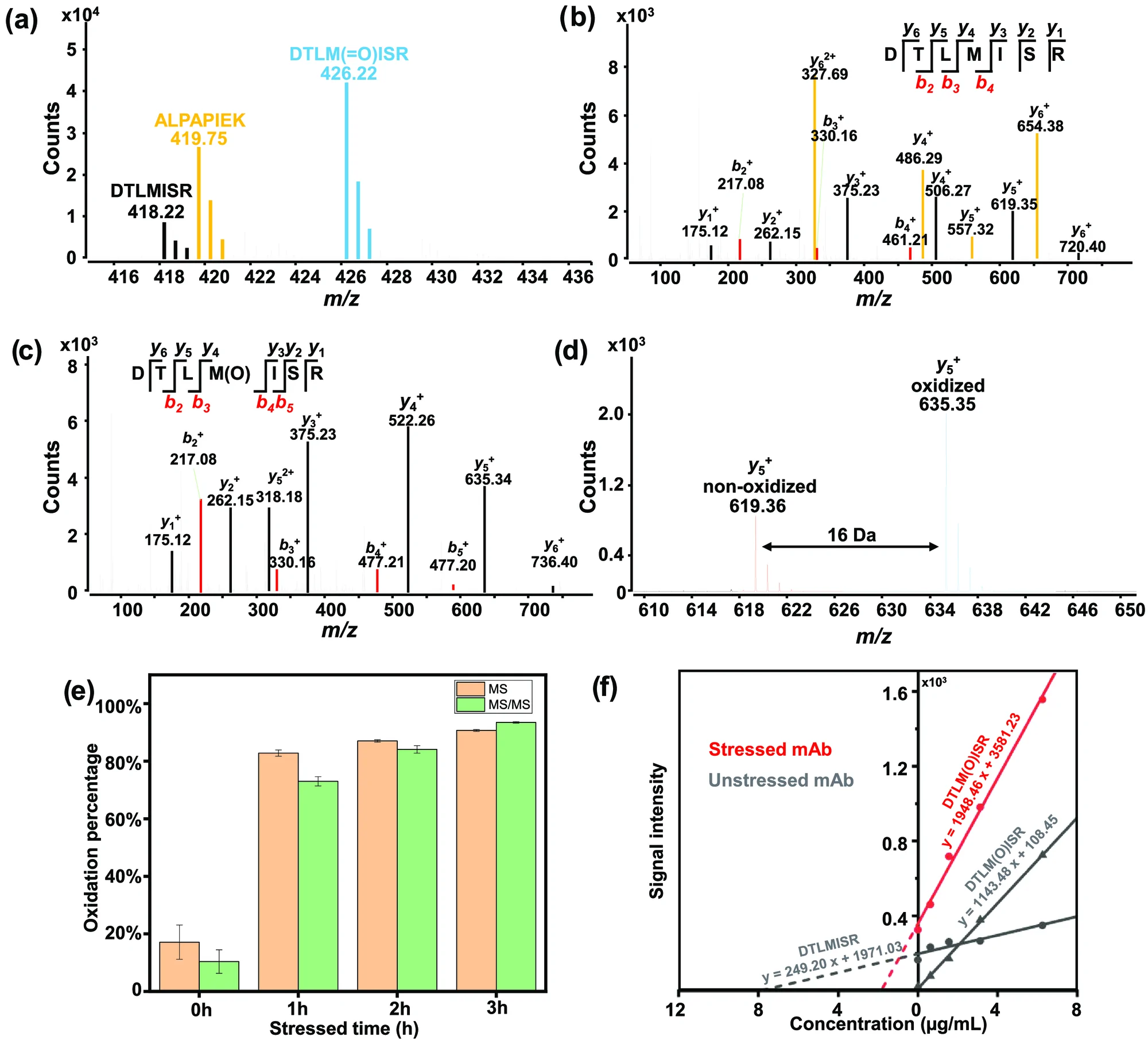
(a) Zoomed-in MS spectrum of microdroplet-digested NIST mAb stressed by hydrogen peroxide showing ions of peptide DTLMISR and its oxidation product DTLM­(O)­ISR as well as ALPAPIEK (in yellow). (b) Target MS/MS spectrum of +2 ion of peptide DTLMISR (*m*/*z* 418.22) with an isolation window of 4 Da; identified *y* and *b* ions were labeled in black and red, respectively; the peaks in yellow were fragment ions from the +2 ion of ALPAPIEK, which was cofragmented with DTLMISR ion. (c) Target MS/MS spectrum of +2 ion of peptide DTLM­(O)­ISR (*m*/*z* 426.22) with an isolation window of 4 Da, identified *y* and *b* ions were labeled in black and red, respectively. (d) Overlapped MS/MS spectrum showing two *y*_5_ fragment ions (differed by 16 Da) generated from CID from +2 ions of DTLMISR (*m*/*z* 418.22) and DTLM­(O)­ISR (*m*/*z* 426.22). (e) Bar graph showing the methionine oxidation percentages for stressed vs nonstressed NIST mAb under different stressing conditions calculated by eq 2 utilizing either MS data (i.e., the intensities of +2 ions of DTLMISR and DTLM­(O)­ISR) and MS/MS data (i.e., *y*_5_ fragment ions from CID of +2 ions of DTLMISR and DTLM­(O)­ISR). (f) Absolute quantification of the intact peptide DTLMISR and the oxidized DTLM­(O)­ISR peptide with the standard addition method.

Relative quantification of DTLMISR oxidation can be straightforward and can be obtained by online microdroplet digestion of the antibody undergoing different stressing conditions, where the peak intensities of *m*/*z* 418.22 and *m*/*z* 426.22 were compared using eq 2. As shown in Figure 3e, the bar graph demonstrates the methionine oxidation percentage for peptide DTLMISR based on both MS and MS/MS quantification following eq 2; a similar oxidation percentage was generated from MS and MS/MS measurement because no overlapping ions were detected with *m*/*z* 418.22 and *m*/*z* 426.22. Based on the MS result, about 17% peptide was oxidized at 0 h, which was higher than the 4.6% oxidation shown in Figure S6 because the mAbs were prepared in 0.1% H_2_O_2_, which oxidized the mAb during sample preparation. However, relative quantification is valid only when the oxidized and nonoxidized peptides exhibit similar ionization efficiencies; otherwise, the quantification result can be biased. To obtain accurate oxidation levels, absolute quantification of oxidized and nonoxidized DTLMISR was therefore performed using a standard addition approach. Known concentrations of synthetic DTLM­(O)­ISR were spiked into hydrogen peroxide-stressed or nonstressed NIST mAbs, followed by online microdroplet digestion. Standard addition curves were generated by plotting the DTLM­(O)­ISR ion intensity (*m*/*z* 426.22) against its spiked concentration. As shown in Figure 3f, the standard addition curve for 1 h-stressed NIST mAb was *y* = 1948.46 *x* + 3581.23 (*R* ^2^ = 0.999), corresponding to an absolute oxidized peptide concentration of 1.83 μg/mL (2.1 × 10^–6^ mol/L). For nonstressed NIST mAb, the curve *y* = 1143.48 *x* + 108.45 (*R* ^2^ = 0.997, Figure 3f) yielded an oxidized peptide concentration of 0.095 μg/mL (1.1 × 10^–7^ mol/L). Standard addition was also performed for the nonoxidized DTLMISR peptide by spiking synthetic DTLMISR into nonstressed NIST mAb. The resulting curve, *y* = 249.20 *x* + 1971.03 (*R* ^2^ = 0.94, Figure 3f), gave an initial DTLMISR concentration of 7.9 μg/mL (9.2 × 10^–6^ mol/L). Based on the ratio of the oxidized peptide mole to the sum of intact and oxidized peptide moles (eq 3), approximately 22% of the peptide was oxidized after 1 h of oxidative stress, whereas only ∼1.2% oxidation was observed in the nonstressed sample.

Based on the standard addition curves, the oxidized peptide DTLM­(O)­ISR exhibited a substantially higher slope than the nonoxidized DTLMISR, as shown in Figure 2f. Specifically, the slope for DTLMISR of unstressed antibody was 249.2, while the slope for DTLM­(O)­ISR of unstressed antibody was 1143.48 (measured from the same microdroplet digestion conditions as DTLMISR), representing a 4.6-fold increase. Because the MS signal intensity is proportional to both the analyte concentration and ionization efficiency, a higher signal at the same concentration indicates enhanced ionization efficiency. The 4.6 times higher slope of DTLM­(O)­ISR indicates enhanced ionization efficiency of 4.6 times of the oxidized peptide in comparison to DTLMISR, which can lead to biased or inaccurate relative quantification using eq 2 because ionization efficiency differences are not properly corrected. Consistent with this observation, the oxidation level determined by relative quantification using eq 2 (Figure S6) was 4.67%; after correction for ionization efficiency by dividing by 4.6, the oxidation level was 1.01%, in close agreement with the 1.2% oxidation calculated by eq 3. Similarly, for the antibody stressed for 1 h, the oxidation level was initially calculated as 82% by relative quantification (Figure 3e); after ionization efficiency correction, this value decreased to 17.8%, which is close to the aforementioned 22% oxidation level obtained by absolute quantification using eq 3.

### Absolute Quantification of Antibody

Currently, bottom-up LC-MS is the most widely used approach for antibody quantification in the biopharmaceutical industry.^27^ In this workflow, the antibody is enzymatically digested into peptides, and surrogate peptides are selected to represent the entire molecule. A stable isotope-labeled version of this peptide is added as an internal standard for absolute quantitation of the antibody peptide (and thereby antibody) by comparing the unlabeled-to-labeled peptide peak ratios against the spiked amount of the labeled standard. However, bottom-up quantification has several limitations, including challenges in surrogate peptide selection, variability in digestion efficiency, sample loss during preparation, and PTMs such as deamidation or oxidation that alter peptide yield and quantification. In contrast, microdroplet digestion offers simpler sample preparation, reduced sample loss, and fewer PTM-related artifacts. In this work, we achieved absolute antibody quantification using an online microdroplet digestion approach after spiking the target antibody with a heavy isotope-labeled antibody internal standard.

In our experiment, a target light Bevacizumab mAb was spiked with a heavy isotope-labeled Bevacizumab mAb in a 1:1 mass ratio (molar concentration ratio: 0.994:1). As shown in Figure 4a, ten pairs of heavy and light peptides were identified after microdroplet digestion of the mixed antibodies, each displaying peak heights close to a 1:1 ratio. For example (Figure 4b), the signal of the proteotypic peptide ALPAPIEK was successfully detected with ALPAPIEK-light (*m*/*z* 838.50) and ALPAPIEK-heavy (*m*/*z* 846.51) differed by an 8 Da mass shift due to the [^13^C_6_, ^15^N_2_]-Lys label. Peptide ALPAPIEK is frequently used in the bioanalysis of antibodies and anti-idiotypic antibodies due its high ionization efficiency and robust fragment ions that result from various LC-MS/MS analysis.^44,45^ In online microdroplet digestion of light and heavy Bevacizumab mAb, the MS1 peak heights were 6.0 × 10^3^ counts for ALPAPIEK-heavy and 5.8 × 10^3^ counts for ALPAPIEK-light, differing by only 3.3%. Targeted MS/MS was performed on both ions (4 Da isolation window), producing the *y*_4_ fragment ions at *m*/*z* 486.29 (light) and 494.30 (heavy, Figure 4c), also showing a near 1:1 peak height ratio. We next performed online microdroplet digestion for quantification; mixtures of heavy and light antibodies were prepared at increasing ratios of heavy/light Bevacizumab mAbs. As shown in Figure 4d, a 2:1 heavy:light mixture produced a corresponding 2:1 peak intensity ratio for ALPAPIEK in the MS spectrum, and the same 2:1 ratio heavy/light *y*_4_ product ions (Figure 4e), confirming the robustness of microdroplet digestion and feasibility for quantitation. A calibration curve was then generated from online microdroplet digestion by correlating the ALPAPIEK heavy-to-light peak intensity ratio with the injected amount of the labeled antibody. The resulting curve (*y* = 0.073x + 0.081) exhibited excellent linearity (*R* ^2^ = 0.99) with 1/*x* weighting. The LOD and LOQ were calculated to be 1.2 and 4.1 ng, respectively, based on three times and ten times the standard deviation of the 6.25 ng injection point (0.03) divided by the calibration slope (0.073, shown in Table S7). Based on the calibration curve, when *y* = 1, it provides the quantity of the target light antibody to be 12.58 ng, which differed only by 0.6% from the theoretical injection amount of 12.5 ng, demonstrating the high accuracy of the method.

**Figure 4:**
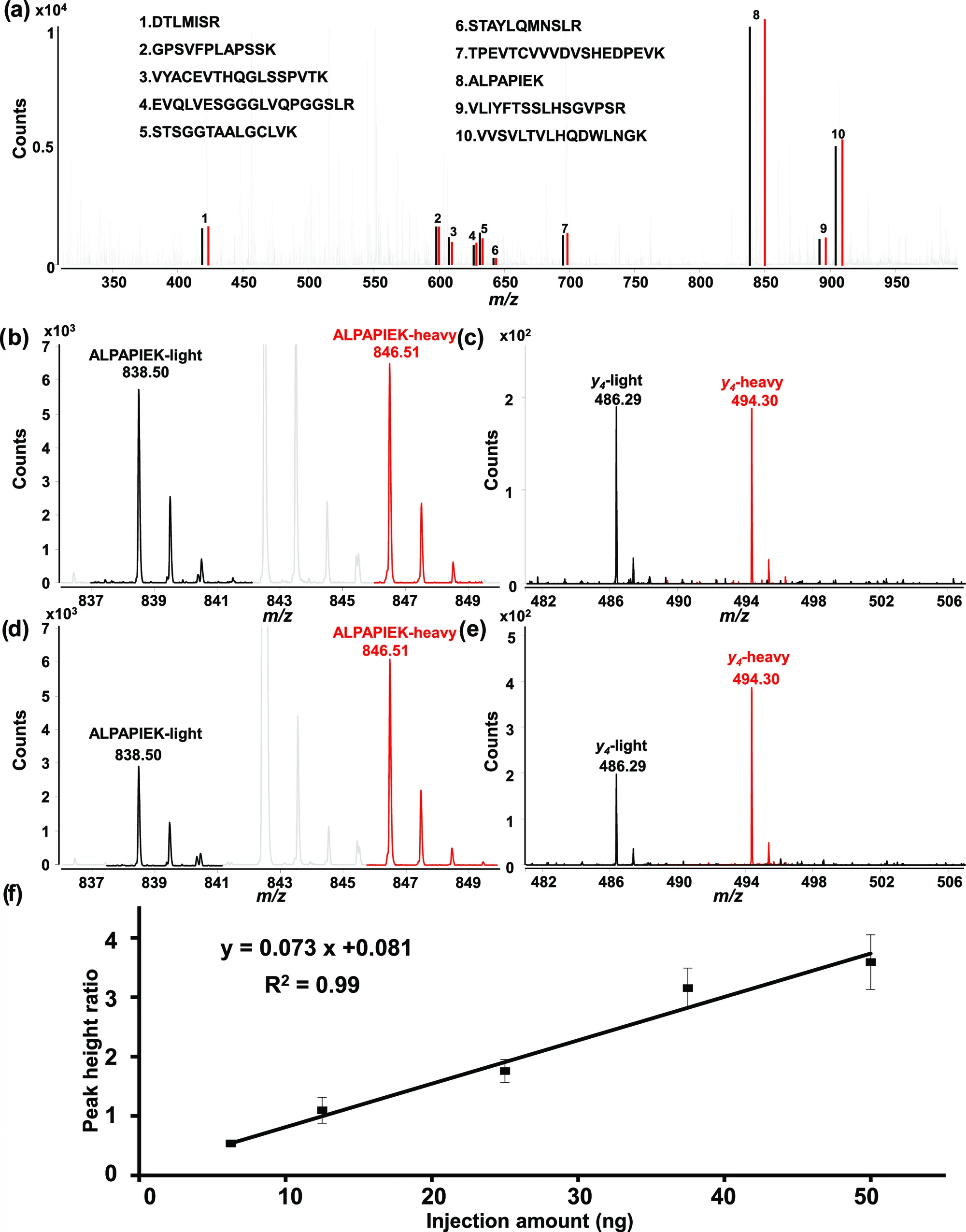
(a) MS spectrum showing online microdroplet digestion of a mixture of 1:1 light and heavy isotope-labeled Bevacizumab mAbs; 10 pairs of light (black) and heavy (red) peptide ions were identified, as highlighted; (b) MS spectrum of heavy and light ALPAPIEK peptide ion (*m*/*z* 838.50 for light, and *m*/*z* 846.51 for heavy) generated by online microdroplet digestion of 1:1 ratio mixed heavy and light Bevacizumab mAbs; (c) MS spectrum of *y*_4_ ions produced by target MS/MS of *m*/*z* 838.50 and 846.51 from 1:1 ratio mixed heavy and light Bevacizumab mAbs; (d) MS spectrum of heavy and light ALPAPIEK peptide ions generated by online microdroplet digestion of 2:1 ratio mixed heavy and light Bevacizumab mAbs; (e) MS spectrum of *y*_4_ ions produced by target MS/MS of *m*/*z* 838.50 and 846.51 from 2:1 ratio mixed heavy and light Bevacizumab mAbs; and (f) calibration curve of the peak height ratio of heavy *y*_4_ over the light *y*_4_ of ALPAPIEK peptide ion against the injection amount of heavy Bevacizumab mAb.

One additional peptide TPEVTCVVVDVSHEDPEVK (Figure S8) was also used for quantification using the same online microdroplet digestion and targeted MS/MS acquisition. As shown in Figure S8, the calibration curve for peptide TPEVTCVVVDVSHEDPEVK is *y* = 0.073*x* – 0.0046 (*R* ^2^ = 0.99). Again, it shows good linearity. When *y* = 1, it provides the quantity of the target light antibody to be 13.76 ng, which shows 10.0% quantitation error and a reasonably good quantitation accuracy, in comparison to the injection amount of 12.5 ng. These results showed that our microdroplet digestion enables fast quantitation of the antibody with simple spiking of a heavy isotope-labeled internal standard.

## Conclusions

In this work, we demonstrated an ultrafast and automated antibody characterization and quantitation method using online microdroplet trypsin digestion coupled with MS/MS analysis. This approach achieved digestion within milliseconds and sample injection cycle times of ∼2.5 min. In particular, our method provided high sequence coverage and enabled the sensitive detection and accurate quantification of PTMs such as asparagine deamidation (by relative quantitation using MS/MS fragment ions) and methionine oxidation (by absolute quantitation via the standard additional method). Furthermore, the method allowed absolute quantification of antibodies through spiking of an isotope-labeled standard antibody. Compared with conventional in-solution digestion, this approach introduces less deamidation and oxidation during sample preparation, offering a rapid and reliable method for peptide mapping and antibody quantitation. We believe that our automated and rapid microdroplet digestion approach is of high value in the biotherapeutics industry.

## Supplementary Material



## Notes

Information about sample preparation, instrument conditions, data analysis, antibody sequences, and additional MS spectra (PDF)
