# Supplementary material for: Rapid Antibody Structural Characterization and Quantification via Microdroplet Trypsin Digestion
Source: J Am Soc Mass Spectrom. 2026 Feb 15;37(10):2281–91. doi: 10.1021/jasms.5c00457 (PMC13651627; doi:10.1021/jasms.5c00457)
Supplement: Supplementary file 1 [file js5c00457_si_001.pdf]

Supporting information

Rapid Antibody Structural Characterization and Quantification via Microdroplet Trypsin Digestion

Mengyuan Xiao[1], Yongqing Yang[1], Andrew Schladebeck,[1] Jim Lau[2], Michael Knierman[2], Hui Zhao[2], Xi Qiu[2], Karen Luo[2], Harsha P. Gunawardena[3,*], and Hao Chen[1,*]

[1]Department of Chemistry & Environmental Science, New Jersey Institute of Technology, Newark, NJ, 07102, USA, Email: hao.chen.2@njit.edu

[2]Agilent Technologies, Inc., 2850 Centerville Rd, Wilmington, DE, 19807, USA

[3]Johnson & Johnson Innovative Medicine, Johnson & Johnson, 1400 McKean Road, Spring House, PA, 19477, USA Email: hgunawar@ITS.JNJ.com

Scheme S1

Sequence of NIST mAb

NIST mAb light chain

DIQMTQSPSTLSASVGDRVTITCSASSRVGYMHWYQQKPGKAPKLLIYDTSKLASGVPS
RFSGSGSGTEFTLTISSLQPDDFATYYCFQGSGYPFTFGGGTKVEIKRTVAAPSVFIFPPSDE
QLKSGTASVVCLLNNFYPREAKVQWKVDNALQSGNSQESVTEQDSKDSTYSLSSTLTLS
KADYEKHKVYACEVTHQGLSSPVTKSFNRGEC

NIST mAb heavy chain

QVTLRESGPALVKPTQTLTLTCTFSGFSLSTAGMSVGWIRQPPGKALEWLADIWWDDKK
HYNPSLKDRLTISKDTSKNQVVLKVTNMDPADTATYYCARDMIFNFYFDVWGQGTTVT
VSSASTKGPSVFPLAPSSKSTSGGTAALGCLVKDYFPEPVTVSWNSGALTSGVHTFPAVL
QSSGLYSLSSVVTVPSSSLGTQTYICNVNHKPSNTKVDKRVEPKSCDKTHTCPPCPAPELL
GGPSVFLFPPKPKDTLMISRTPEVTCVVVDVSHEDPEVKFNWYVDGVEVHNAKTKPREE
QYNSTYRVVSVLTVLHQDWLNGKEYKCKVSNKALPAPIEKTISKAKGQPREPQVYTLPP
SREEMTKNQVSLTCLVKGFYPSDIAVEWESNGQPENNYKTTPPVLDSDGSFFLYSKLTVD
KSRWQQGNVFSCSVMHEALHNHYTQKSLSLSPGK

Scheme S2

Sequences for light and heavy SigmaMab Bevacizumab:

Heavy labeled amino acids are $^{13}C6$, $^{15}N4$-labeled arginine and $^{13}C6$, $^{15}N2$-labeled lysine, the molecular weight of light Sigma mAb is 146.2K Da, and the molecular weight of heavy Sigma mAb was 147.3 K Da.

SigmaMab Bevacizumab Light Chain:
DIQMTQSPSSLSASVGDRVTITCSASQDISNYLNWYQQKPGKAPKVLIYFTSSLHSGVPSR
FSGSGSGTDFTLTISSLQPEDFATYYCQQYSTVPWTFGQGTKVEIKRTVAAPSVFIFPPSDE
QLKSGTASVVCLLNNFYPREAKVQWKVDNALQSGNSQESVTEQDSKDSTYSLSSTLTLS
KADYEKHKVYACEVTHQGLSSPVTKSFNRGEC

SigmaMab Bevacizumab Heavy Chain:
EVQLVESGGGLVQPGGSLRLSCAASGYTFTNYGMNWVRQAPGKGLEWVGWINTYTGE
PTYAADFKRRFTFSLDTSKSTAYLQMNSLRAEDTAVYYCAKYPHYYGSSHWYFDVWGQ
GTLVTVSSASTKGPSVFPLAPSSKSTSGGTAALGCLVKDYFPEPVTVSWNSGALTSGVHT
FPAVLQSSGLYSLSSVVTVPSSSLGTQTYICNVNHKPSNTKVDKKVEPKSCDKTHTCPPCP
APELLGGPSVFLFPPKPKDTLMISRTPEVTCVVVDVSHEDPEVKFNWYVDGVEVHNAKT
KPREEQYNSTYRVVSVLTVLHQDWLNGKEYKCKVSNKALPAPIEKTISKAKGQPREPQV
YTLPPSREEMTKNQVSLTCLVKGFYPSDIAVEWESNGQPENNYKTTPPVLDSDGSFFLYS
KLTVDKSRWQQGNVFSCSVMHEALHNHYTQKSLSLSPG

Scheme 3. Calculation of the contact time of antibody and trypsin after injection

Tubing volume $= \pi \times r^2 \times$ tube length $= 3.14 \times (0.00635\ \text{cm})^2 \times 70\ \text{cm} = 8.85\ \mu\text{L}$

Flow rate after mixing $= 100\ \mu\text{L/min}$

Time $= 8.85\ \mu\text{L} / 100\ \mu\text{L/min} = 5.3$ sec

Table S1. Programmed injector sequence for autosampler

Function	Parameter
Draw	Draw 0.20 µL from sample
Wash	Wash needle in flushport for 2 s
Draw	Draw 0.20 µL from location (enzyme)
Mix	Mix 1.00 µL from air with default speed for 5 times
Wash	Wash needle in flushport 2 s
Remote	Set remote line for 125 ms
Wait For	Wait for 0.03 min
Inject	Inject

Table S2. Deamidation percentage calculation for peptide GFYPSDIAVEWESNGQPENNYK (5- and 7-days incubation, based on y_6 and y_{10} analysis)

	Deamidation condition		Control condition			Deamidation condition				Control condition			
5 days incubation y6	y peak height	y+1 peak height	y peak height	y+1 peak height	Theoretical I_{y+1}/I_y ratio	Theoretical y+1 without deamidation	Increased y+1 peak height	Deamidation percentage	Average deamidation percentage	Theoretical y+1 without deamidation	Increased y+1 peak height	Deamidation percentage	Average deamidation percentage
Exp.1	54.14	26.16	412.4	192.5		19.49	6.67	11%		148.46	44.04	10%	
Exp.2	110.97	38.48	404.45	162.47	0.36	39.95	-1.47	-1%	7%	145.6	16.87	4%	5%
Exp.3	73.4	34.97	512.26	198.87		26.42	8.55	10%		184.41	14.46	3%	
	Deamidation condition		Control condition			Deamidation condition				Control condition			
5 days incubation y10	y peak height	y+1 peak height	y peak height	y+1 peak height	Theoretical I_{y+1}/I_y ratio	Theoretical y+1 without deamidation	Increased y+1 peak height	Deamidation percentage	Average deamidation percentage	Theoretical y+1 without deamidation	Increased y+1 peak height	Deamidation percentage	Average deamidation percentage
Exp.1	7.1	7.9	55.09	26.45		3.69	4.21	37%		28.65	-2.2	-4%	
Exp.2	8.04	9.02	48.04	44.22	0.52	4.18	4.84	38%	39%	24.98	19.24	29%	15%
Exp.3	6.09	7.57	52.65	41.51		3.17	4.4	42%		27.38	14.13	21%	
	Deamidation condition		Control condition			Deamidation condition				Control condition			
7 days incubation y6	y peak height	y+1 peak height	y peak height	y+1 peak height	Theoretical I_{y+1}/I_y ratio	Theoretical y+1 without deamidation	Increased y+1 peak height	Deamidation percentage	Average deamidation percentage	Theoretical y+1 without deamidation	Increased y+1 peak height	Deamidation percentage	Average deamidation percentage
Exp.1	69.32	31.07	626.57	255.57		24.96	6.11	8%		225.57	30	5%	
Exp.2	81.99	44.8	671.74	263.33	0.36	29.52	15.28	16%	11%	241.83	21.5	3%	4%
Exp.3	70.12	31.79	585	246		25.24	6.55	9%		210.6	35.4	6%	
	Deamidation condition		Control condition			Deamidation condition				Control condition			
7 days incubation y10	y peak height	y+1 peak height	y peak height	y+1 peak height	Theoretical I_{y+1}/I_y ratio	Theoretical y+1 without deamidation	Increased y+1 peak height	Deamidation percentage	Average deamidation percentage	Theoretical y+1 without deamidation	Increased y+1 peak height	Deamidation percentage	Average deamidation percentage
Exp.1	6.86	9.41	92.84	48.42		3.57	5.84	46%		48.28	0.14	0%	
Exp.2	9.32	11.54	72.23	55.18	0.52	4.85	6.69	42%	45%	37.56	17.62	20%	10%
Exp.3	9.61	13.25	68.58	44.58		5	8.25	46%		35.66	8.92	12%	

Table S3. Deamidation percentage calculation for peptide TPEVTCVVVDVSHEDPEVKFNWYVDGVEVHNAK (7-days incubation, based on y_8 and y_{15} analysis)

	Deamidation condition		Control condition			Deamidation condition				Control condition			
7 days incubation y8	y peak height	y+1 peak height	y peak height	y+1 peak height	Theoretical I_{y+1}/I_y ratio	Theoretical y+1 without deamidation	Increased y+1 peak height	Deamidation percentage	Average deamidation percentage	Theoretical y+1 without deamidation	Increased y+1 peak height	Deamidation percentage	Average deamidation percentage
Exp.1	1081.31	511.33	414.04	199.66	0.39	421.7109	89.6191	8%	8.2%	161.4756	38.1844	8%	7.2%
Exp.2	891.19	415.75	384.82	180.02		347.5641	68.1859	7%		150.0798	29.9402	7%	
Exp.3	752.1	375.89	379.92	172.08		293.319	82.571	10%		148.1688	23.9112	6%	
	Deamidation condition		Control condition			Deamidation condition				Control condition			
7 days incubation y15	y peak height	y+1 peak height	y peak height	y+1 peak height	Theoretical I_{y+1}/I_y ratio	Theoretical y+1 without deamidation	Increased y+1 peak height	Deamidation percentage	Average deamidation percentage	Theoretical y+1 without deamidation	Increased y+1 peak height	Deamidation percentage	Average deamidation percentage
Exp.1	224.34	258.2	78.5	55.89	0.92	206.3928	51.8072	19%	8.8%	72.22	-16.33	-26%	6.4%
Exp.2	183.24	158.55	76.7	88.2		168.5808	-10.0308	-6%		70.564	17.636	19%	
Exp.3	156.14	168.13	81.39	66.24		143.6488	24.4812	14%		74.8788	-8.6388	-12%	

Table S4. Deamidation percentage calculation for peptide VVSVLTVLHQDWLNGKEYK (7-days incubation, based on y_9 analysis)

	Deamidation condition		Control condition			Deamidation condition				Control condition			
7 days incubation y9	y peak height	y+1 peak height	y peak height	y+1 peak height	Theoretical I_{y+1}/I_y ratio	Theoretical y+1 without deamidation	Increased y+1 peak height	Deamidation percentage	Average deamidation percentage	Theoretical y+1 without deamidation	Increased y+1 peak height	Deamidation percentage	Average deamidation percentage
Exp.1	140.97	97.12	412.27	265.29		81.7626	15.3574	10%		239.1166	26.1734	6%	
Exp.2	170.84	115.94	356.7	226.27	0.58	99.0872	16.8528	9%	10.0%	206.886	19.384	5%	4.0%
Exp.3	148.2	107.39	426	252		85.956	21.434	13%		247.08	4.92	1%	

Table S5. Deamidation percentage calculation for peptide NQVVLK (7-days incubation, based on b_2 analysis)

	Deamidation condition		Control condition			Deamidation condition				Control condition			
7 days incubation b2	b peak height	b+1 peak height	b peak height	b+1 peak height	Theoretical b_{y+1}/b_y ratio	Theoretical b+1 without deamidation	Increased b+1 peak height	Deamidation percentage	Average deamidation percentage	Theoretical b+1 without deamidation	Increased b+1 peak height	Deamidation percentage	Average deamidation percentage
Exp.1	882	112	1933.47	282.54		88.2	23.8	2.6%		193.347	89.193	4.4%	
Exp.2	573	75	1838.11	269.98	0.1	57.3	17.7	3.0%	3.7%	183.811	86.169	4.5%	4.3%
Exp.3	463.1	73.5	1896.65	271.41		46.31	27.19	5.5%		189.665	81.745	4.1%	

Table S6. Deamidation percentage calculation for peptide SGTASVVCLLNNFYPR (7-days incubation, based on y_5 and y_6 analysis)

	Deamidation condition		Control condition			Deamidation condition				Control condition			
7 days incubation y5	y peak height	y+1 peak height	y peak height	y+1 peak height	Theoretical I_{y+1}/I_y ratio	Theoretical y+1 without deamidation	Increased y+1 peak height	Deamidation percentage	Average deamidation percentage	Theoretical y+1 without deamidation	Increased y+1 peak height	Deamidation percentage	Average deamidation percentage
Exp.1	122.3	49.44	1049.26	464.93		44.028	5.412	4.2%		377.7336	87.1964	7.7%	
Exp.2	131.87	59.08	955.82	459.84	0.36	47.4732	11.6068	8.1%	6.5%	344.0952	115.7448	10.8%	7.5%
Exp.3	125.86	55.24	985.22	396.03		45.3096	9.9304	7.3%		354.6792	41.3508	4.0%	
	Deamidation condition		Control condition			Deamidation condition				Control condition			
7 days incubation y6	y peak height	y+1 peak height	y peak height	y+1 peak height	Theoretical I_{y+1}/I_y ratio	Theoretical y+1 without deamidation*	Increased y+1 peak height	Deamidation percentage	Average deamidation percentage	Theoretical y+1 without deamidation*	Increased y+1 peak height	Deamidation percentage	Average deamidation percentage
Exp.1	360.04	198.44	3468.44	1701.52		144.016	54.424	13.1%		1387.376	314.144	8.3%	
Exp.2	363.02	199.46	3253	1590	0.4	145.208	54.252	13.0%	13.6%	1301.2	288.8	8.2%	8.6%
Exp.3	317.26	181.94	3036.88	1533.97		126.904	55.036	14.8%		1214.752	319.218	9.5%	

Table S7. Absolute quantification of light Bevacizumab mAb using peptide ALPAPIEK

	Ratio of I_{y+8}/I_y					LOD (ng)	LOQ (ng)
Injected amount (MSQC20 ng)	Run-1	Run-2	Run-3	Average ratio	STDEV	1.2	4.1
6.25	0.53	0.51	0.58	0.54	0.03		
12.50	0.97	1.01	1.37	1.10	0.22		
25.00	1.83	1.55	1.92	1.76	0.19		
37.50	3.55	2.91	3.05	3.15	0.34		
50.00	4.09	3.44	3.20	3.59	0.46		

LOD = (3×STDEV at 6.25 ng injection point)÷slope of calibration curve (Figure 4f)

= (3×0.03)÷0.073

= 1.2 ng

LOQ = (10×STDEV at 6.25 ng injection point)÷slope of calibration curve (Figure 4f)

= (10×0.03)÷0.073

= 4.1 ng

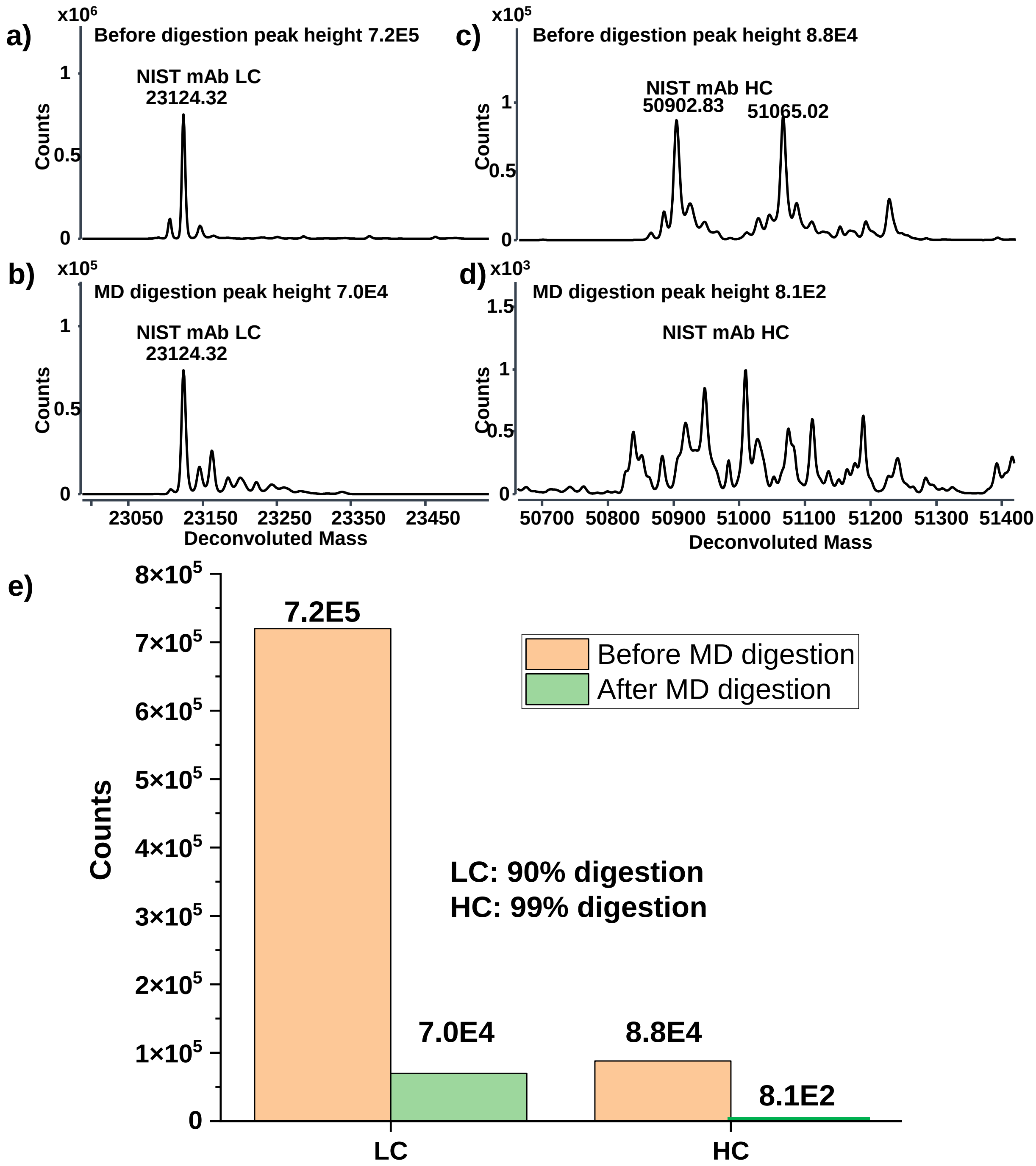

Figure S1. a) Deconvoluted spectra of NIST antibody light chain a) before microdroplet digestion and b) after microdroplet digestion; Deconvoluted spectra of NIST antibody heavy chain before c) online microdroplet digestion and d) after online microdroplet digestion; e) Bar graph comparing the online microdroplet digestion efficiencies of NIST mAb light chain (LC) and heavy chain (HC).

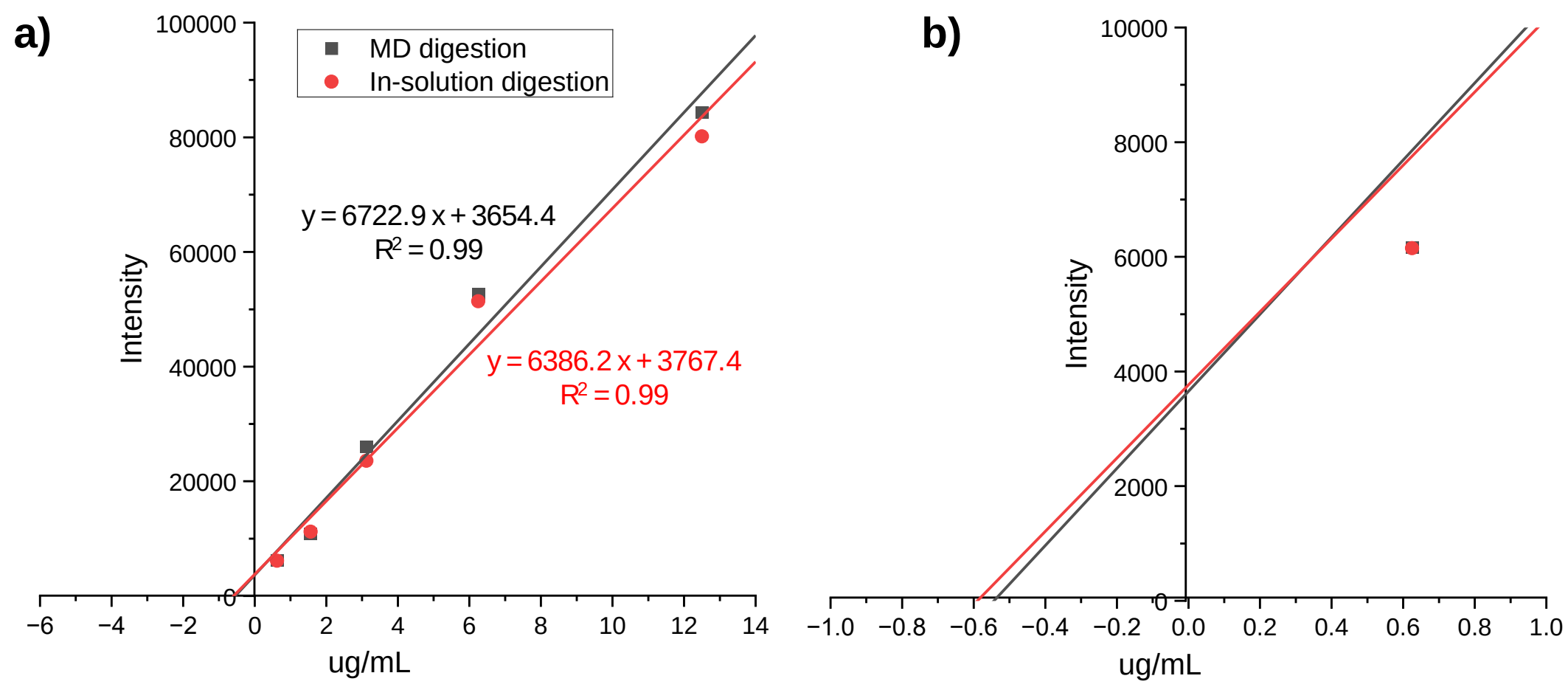

Figure S2. a) Digestion efficiency comparison between online microdroplet digestion and in-solution digestion with a standard-addition method analysis by spiking NIST antibody with different concentrations of DTLMISR peptide. The result suggests similar digestion efficiency of online microdroplet digestion vs. in-solution digestion of NIST antibody. b) zoomed-in calibration curve showing X-intercepts

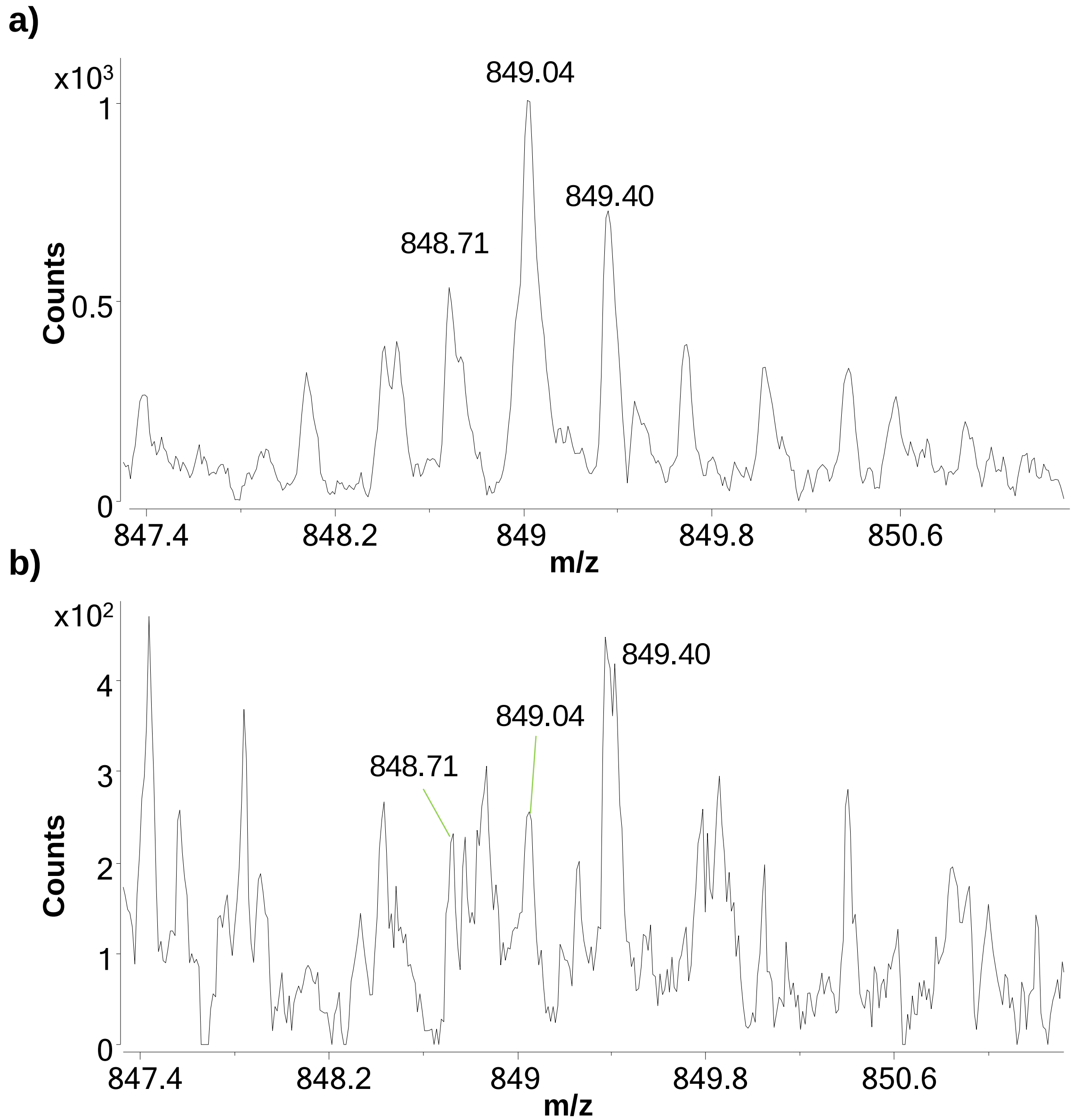

Figure S3. MS spectra of peptide GFYPSDIAVEWESNGQPENNYK generated by microdroplet digestion of NIST antibody incubated in a) deionized water for 5 days, and b) pH 8 Tris buffer for 5 days.

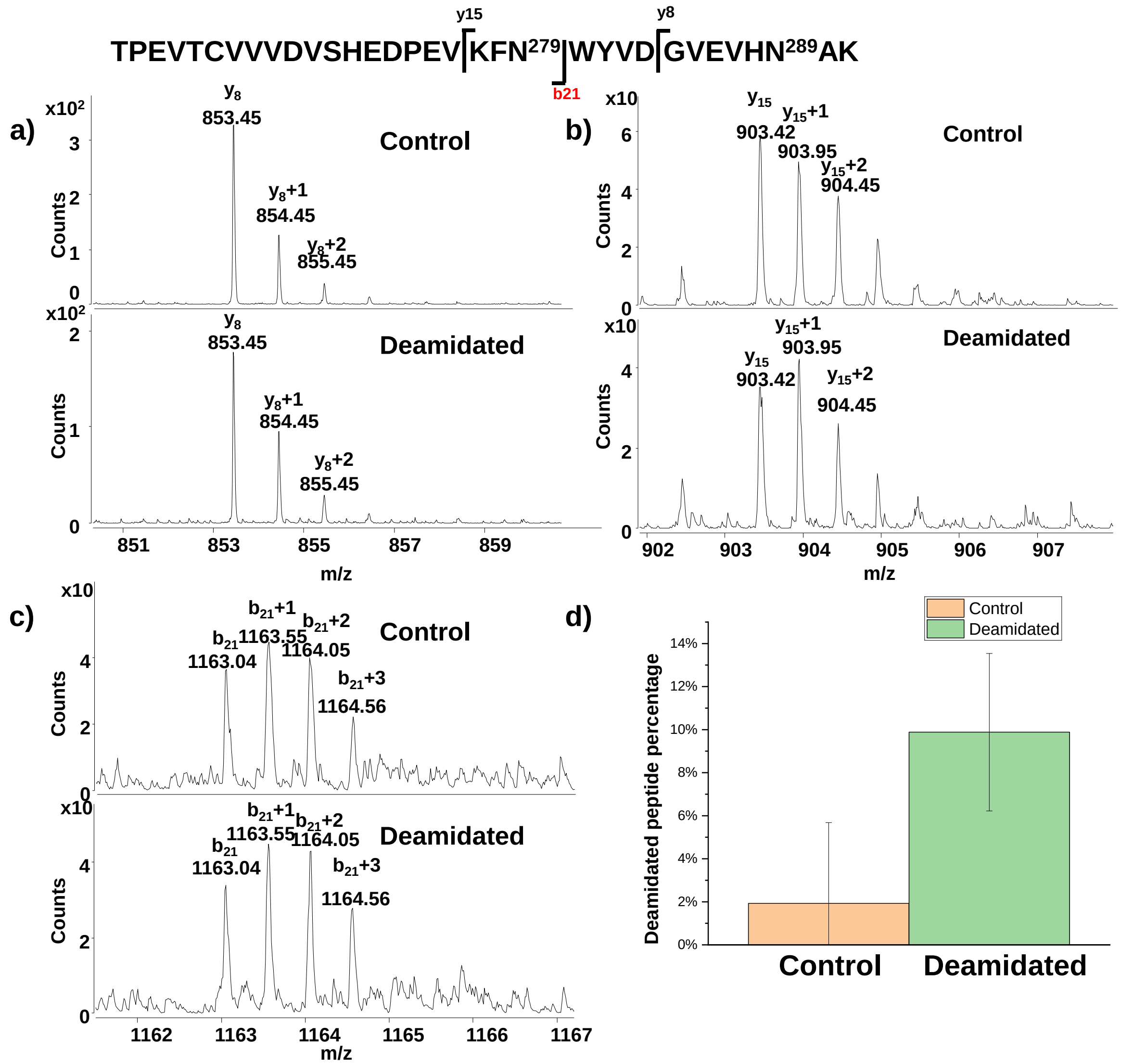

Figure S4. MS/MS spectra showing a) y_8, b) y_{15} and c) b_{21} fragment ions from CID of +3 ion (*m/z* 1247.60) of TPEVTCVVVDVSHEDPEVKFN^{279}WYVDGVEVHN^{289}AK generated from online microdroplet digested NIST mAb after incubation in deionized water (Control) or in pH 8 Tris buffer (Deamidation condition); d) Deamidation percentages of TPEVTCVVVDVSHEDPEVKFN^{279}WYVDGVEVHN^{289}AK after 7 days incubations in deionized water (Control) or in pH 8 Tris buffer (Deamidation condition), based on calculation using y_8 fragment ion.

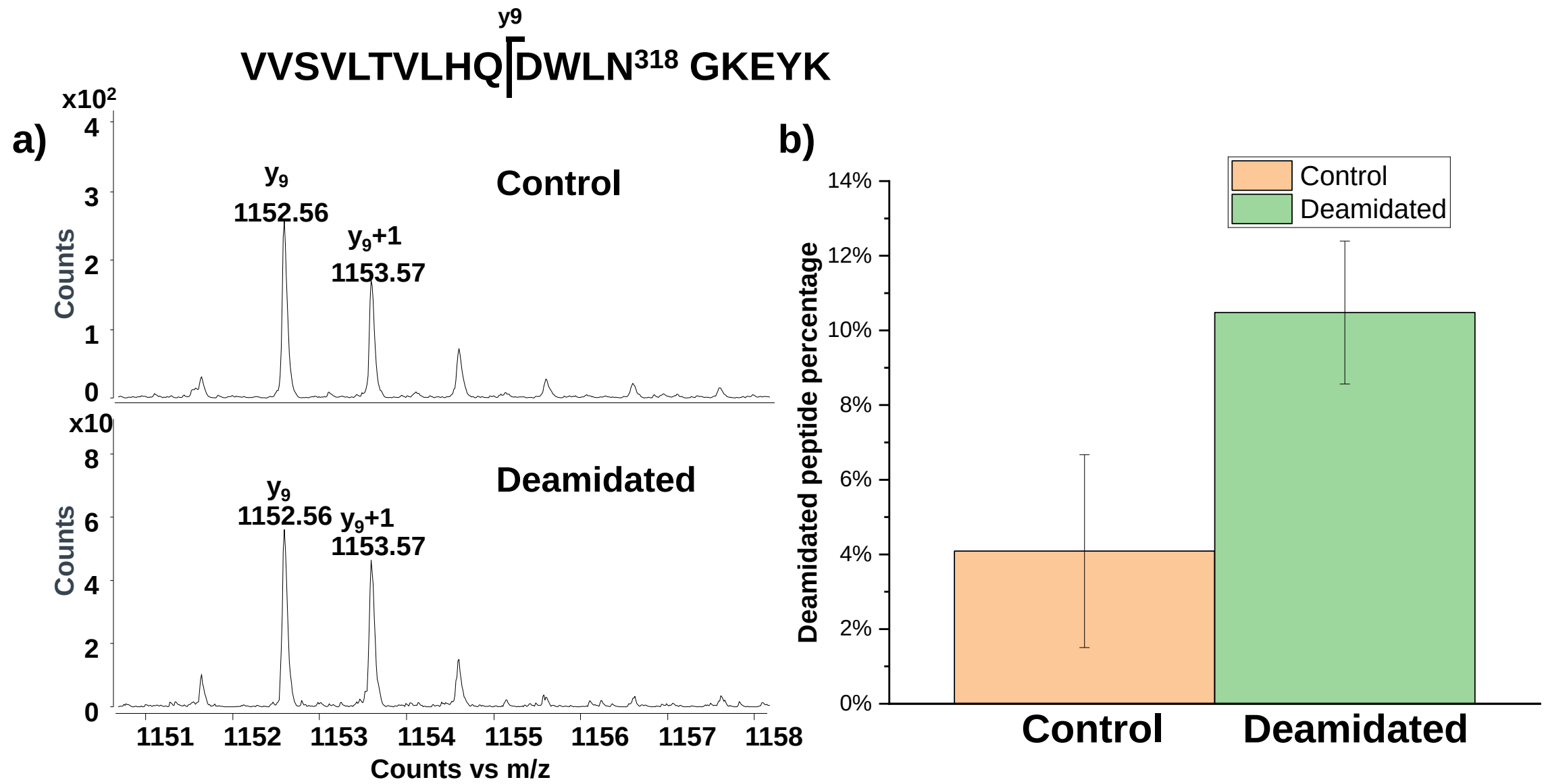

Figure S5. a) MS/MS spectrum showing y_9 fragment ion from CID of +3 ion (m/z 743.40) of VVSVLTVLHQDWLN^{318}GKEYK peptide generated from online microdroplet digested NIST mAb, after incubation in deionized water (Control) or pH 8 Tris buffer (Deamidation condition); b) Deamidation percentages of VVSVLTVLHQDWLN^{318}GKEYK after 7-days incubation in deionized water (Control) or in pH 8 Tris buffer (Deamidation condition), based on calculation using y_9 fragment ion.

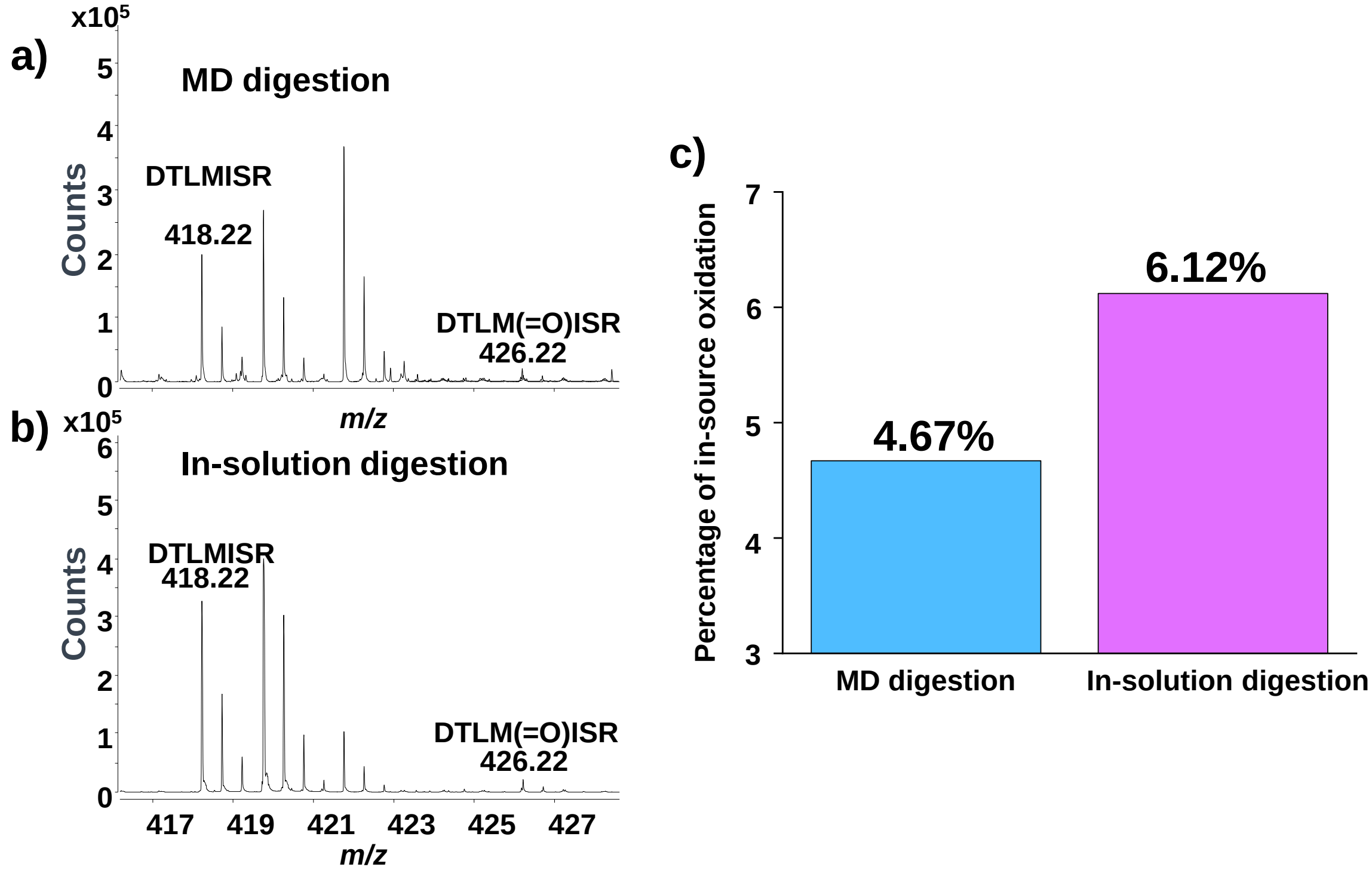

Figure S6. Oxidation modification of DTLMISR peptide compared between a) microdroplet digestion and b) in-solution digestion; c) Bar graph compared the percentages of the oxidized DTLMISR peptide, suggesting a higher amount of oxidation for in-solution digestion.

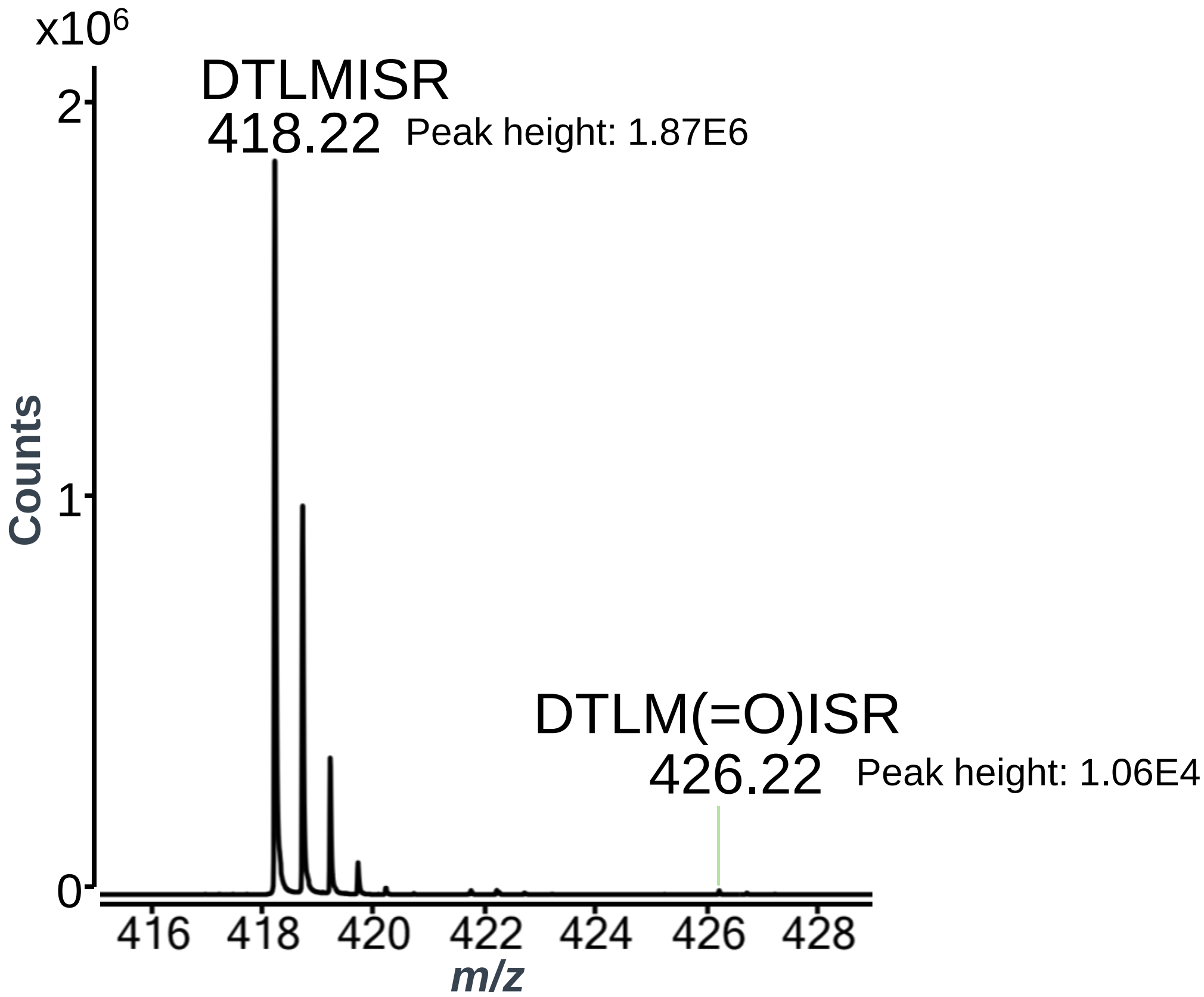

Figure S7. Flow injection of the standard DTLMISR peptide with the same acquisition method as online microdroplet digestion, showing less than 1% of in-source oxidation of peptide DTLMISR.

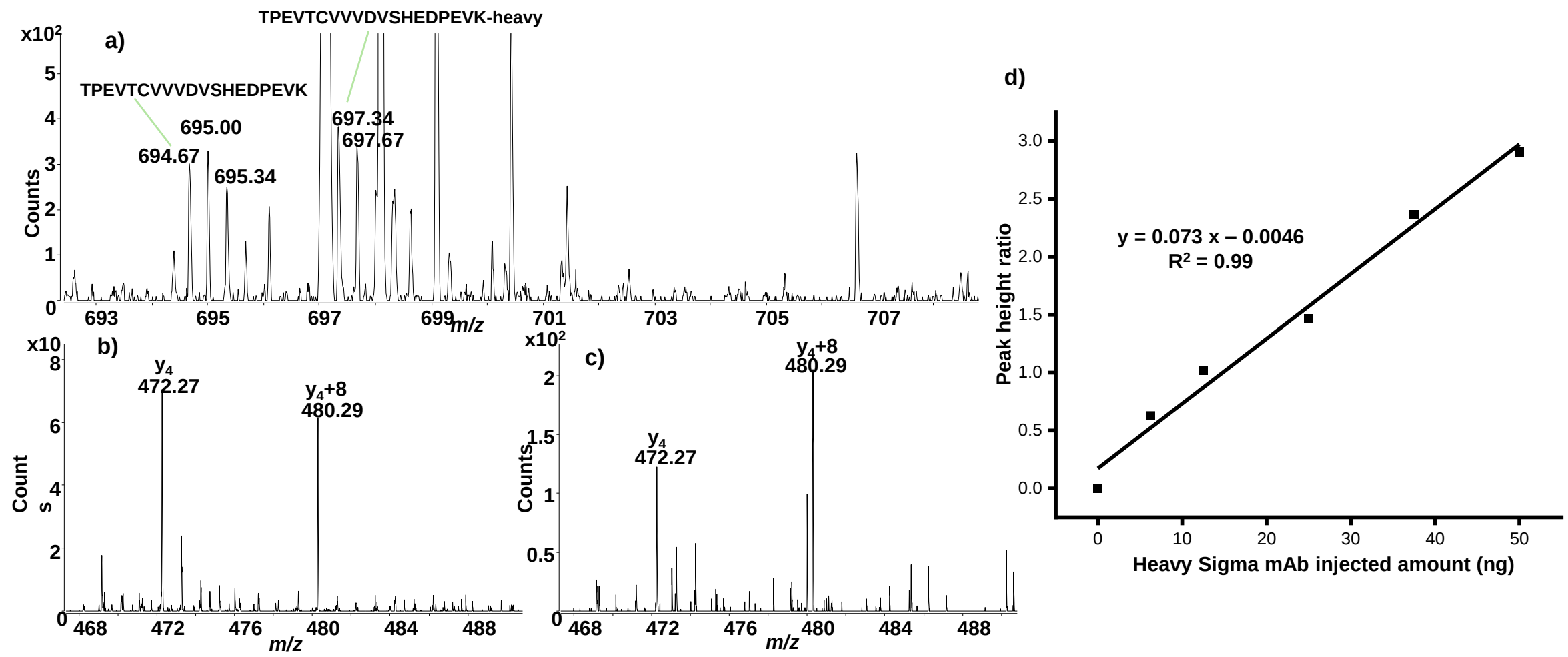

Figure S8. a) MS spectrum showing +3 ions of TPEVTCVVVDVSHEDPEVK and TPEVTCVVVDVSHEDPEVK-heavy peptide generated from online microdroplet digestion of a mixture of heavy and light Bevacizumab mAbs (mixed by 1:1 mass ratio); b) MS/MS spectrum showing two *y4* fragment ions (differed by 8 Da) generated from CID of the TPEVTCVVVDVSHEDPEVK ion (*m/z* 694.67) and TPEVTCVVVDVSHEDPEVK -heavy ion (*m/z* 697.34), respectively. The heavy and light Bevacizumab mAbs were mixed by 1 to 1 mass ratio for microdroplet digestion; c) MS/MS spectrum showing two *y4* fragment ions (differed by 8 Da) generated from CID of the TPEVTCVVVDVSHEDPEVK ion (*m/z* 694.67) and TPEVTCVVVDVSHEDPEVK -heavy ion (*m/z* 697.34), respectively. The heavy and light Bevacizumab mAbs were mixed by 2 to 1 mass ratio for microdroplet digestion; d) Calibration curve showing the linear relation of the peak height ratio of the two *y4* fragment ions (heavy *y4* vs. light *y4*) to the injection amount of heavy Bevacizumab mAb.
